# Effectiveness of Mobile Health–Based Gamification Interventions for Improving Physical Activity in Individuals With Cardiovascular Diseases: Systematic Review and Meta-Analysis of Randomized Controlled Trials

**DOI:** 10.2196/64410

**Published:** 2025-01-24

**Authors:** Tianzhuo Yu, Monica Parry, Tianyue Yu, Linqi Xu, Yuejin Wu, Ting Zeng, Xin Leng, Qian Tong, Feng Li

**Affiliations:** 1 School of Nursing Jilin University Changchun China; 2 Lawrence Bloomberg Faculty of Nursing University of Toronto Toronto, ON Canada; 3 Faculty of Medicine and Life Sciences University of Hasselt Diepenbeek Belgium; 4 Department of Cardiovascular Medicine Bethune First Hospital of Jilin University Changchun China

**Keywords:** cardiovascular diseases, digital health, mobile health, gamification, exercise, physical activity, systematic review, meta-analysis

## Abstract

**Background:**

Gamification refers to using game design elements in nongame contexts. Promoting physical activity (PA) through gamification is a novel and promising avenue for improving lifestyles and mitigating the advancement of cardiovascular diseases (CVDs). However, evidence of its effectiveness remains mixed.

**Objective:**

This systematic review and meta-analysis aimed to evaluate the efficacy of gamification interventions in promoting PA during short-term and follow-up periods in individuals with CVDs and to explore the most effective game design elements.

**Methods:**

A comprehensive search of 7 electronic databases was conducted for randomized controlled trials published in English from January 1, 2010, to February 3, 2024. Eligible studies used mobile health–based gamification interventions to promote PA or reduce sedentary behavior in individuals with CVDs. In total, 2 independent reviewers screened the retrieved records, extracted data, and evaluated the risk of bias using the RoB 2 tool. Discrepancies were resolved by a third reviewer. Meta-analyses were performed using a random-effects model with the Sidik-Jonkman method adjusted by the Knapp-Hartung method. Sensitivity analysis and influence analysis examined the robustness of results, while prediction intervals indicated heterogeneity. A meta-regression using a multimodel inference approach explored the most important game design elements. Statistical analyses were conducted using R (version 4.3.2; R Foundation for Statistical Computing).

**Results:**

In total, 6 randomized controlled trials were included. Meta-analysis of 5 studies revealed a small effect of gamification interventions on short-term PA (after sensitivity analysis: Hedges *g*=0.32, 95% CI 0.19-0.45, 95% prediction interval [PI] 0.02-0.62). Meta-analysis of 3 studies found the maintenance effect (measured with follow-up averaging 2.5 months after the end of the intervention) was small (Hedges *g*=0.20, 95% CI 0.12-0.29, 95% PI –0.01 to 0.41). A meta-analysis of 3 studies found participants taking 696.96 more steps per day than the control group (95% CI 327.80 to 1066.12, 95% PI –121.39 to 1515.31). “Feedback” was the most important game design element, followed by “Avatar.”

**Conclusions:**

This meta-analysis demonstrates that gamification interventions effectively promote PA in individuals with CVD, with effects persisting beyond the intervention period, indicating they are not merely novel effects caused by the game nature of gamification. The 95% PI suggests that implementing gamification interventions in similar populations in the future will lead to actual effects in promoting PA in the vast majority of cases. However, the limited number of included studies underscores the urgent need for more high-quality research in this emerging field.

**Trial Registration:**

PROSPERO CRD42024518795; https://www.crd.york.ac.uk/prospero/display_record.php?RecordID=518795

## Introduction

### Background

Cardiovascular diseases (CVDs) are a group of disorders affecting the heart and blood vessels that significantly contribute to premature mortality and escalating health care expenses [1,2]. According to the Global Burden of Disease 2021 Study, the global prevalence of CVD cases has reached 612 million [3]. Regular physical activity (PA) is widely recognized as one of the most effective lifestyle interventions for managing CVD risk factors, slowing disease progression, and reducing CVD-related mortality [4]. The World Health Organization guidelines on PA recommend that individuals with CVD engage in 150-300 minutes of moderate-intensity PA, 75-150 minutes of vigorous-intensity PA, or an equivalent combination of moderate-to-vigorous intensity PA per week [5]. Moreover, as self-reported sedentary behavior (SB) is independently associated with an increased risk of CVD, irrespective of PA levels [6,7], the guidelines emphasize minimizing sedentary time and replacing it with any intensity of PA to achieve health benefits [5].

Adherence in the health domain is defined as “the extent to which a person’s behavior—taking medication, following a diet, and/or executing lifestyle changes—corresponds with agreed recommendations from a health care provider” [8]. Despite the well-documented benefits of PA, studies indicate that many individuals with CVD demonstrate poor adherence, often failing to meet the PA levels recommended by guidelines [9,10]. Promoting health behavior change and improving PA adherence in individuals with CVD is challenging due to various factors, including lack of motivation or self-efficacy, cognitive or physical limitations, and other barriers [11]. In response, there is a growing call to shift the focus of PA from its health-promoting utility to emphasizing the personal experience [12]. Since emotion plays an essential role in driving behavior, creating opportunities for individuals with CVD to “feel good” while engaging in PA and building a connection between pleasurable feelings and the activity may provide an effective strategy to enhance PA adherence.

Gamification, defined as the use of game design elements in nongame contexts [13], has shown promise in promoting adherence to PA among adults [14], offering a novel approach to modifying health behaviors [15]. Notably, studies have demonstrated that gamification is not only enjoyable for individuals with CVD but also increases the pleasure of engaging in PA [16] and supports maintaining a high level of adherence [17]. In recent years, gamification has been increasingly integrated into mobile health (mHealth), which leverages mobile computing and communication technologies—such as mobile phones and wearable sensors—to deliver medical services and information [18]. With its powerful capabilities for sensing, processing, storing, and displaying data, mHealth enables continuous tracking and collecting of PA-related information, extending gamification into daily health behaviors [15,19]. The combination of gamification and mHealth is mutually reinforcing, providing opportunities to enhance the quality and experience of CVD care [20].

Although gamification has been studied as a strategy to improve PA- and health-related outcomes in individuals with CVD [16,17,21], no systematic review on this specific topic has been published to date. Existing systematic reviews have primarily examined the application of gamification in populations with conditions such as hypertension, excess weight, and diabetes [22,23]. However, these reviews did not exclusively focus on secondary prevention or PA outcomes in individuals with established CVD. Notably, these reviews highlighted the necessity for high-quality studies [23]. They emphasized the need for randomized controlled trials (RCTs) to identify effective and acceptable gamification interventions for the self-management of CVD [22]. Furthermore, evidence regarding the effectiveness of mHealth-based gamification interventions in improving PA among individuals with CVD remains inconclusive. Given the growing body of RCTs in this area, a systematic review and meta-analysis focused on secondary prevention of CVD appears timely and warranted to address these gaps.

### Objectives

This study aims to quantify the effects of gamification interventions on PA in individuals with established CVD. Effective gamification outcomes should extend beyond short-term novelty effects measured immediately after the intervention to include lasting impacts assessed at the end of a predefined follow-up period [24]. Therefore, we evaluated the effects of PA both postintervention and at the end of follow-up periods as defined by the included studies. Additionally, prediction intervals (PIs) were reported alongside CIs in the meta-analyses. PIs provide an estimate of the range within which future individual observations will likely fall, offering insights often overlooked [25]. IntHout et al [26] demonstrated that implementing PIs in over 400 published meta-analyses led to completely opposite effects in more than 20% of cases. Last, a fundamental limitation of current gamification research is the inability to determine which game design elements contribute most to its efficacy [16,22]. This highlights the need for further research to identify and isolate the most active and effective gamification elements [27].

This systematic review and meta-analysis aim to address key research gaps by (1) assessing the impact of gamification interventions on PA in individuals with CVD, (2) evaluating the follow-up effects of these interventions, (3) reporting the PIs to estimate the potential intervention effects in future studies, and (4) determining the most effective game design element for influencing PA behaviors.

## Methods

This review follows the PRISMA (Preferred Reporting Items for Systematic Reviews and Meta-Analyses) 2020 statement [28]. The methods were preregistered with PROSPERO (International Prospective Register of Systematic Reviews, registration number CRD42024518795, registration on February 29, 2024).

### Eligibility Criteria

Eligible studies met the following criteria:

1. Participants: Adults aged 18 years or older with CVD, such as coronary heart disease, peripheral artery disease (PAD), as well as heart attacks and strokes.
2. Interventions: Included studies focused on gamification interventions delivered via mHealth, aiming to improve PA. These interventions included game design elements such as points, levels, challenges, progress bars, leaderboards, rewards, collaboration, social support, and avatars. Gamification and serious games should be clearly distinguished. Therefore, interventions involving virtual reality, active video games, or motion-sensing technologies (eg, Xbox 360, Kinect, Wii) were excluded. mHealth incorporated at least one of the following components: wearable devices, portal websites, smartphone applications, or messaging services.
3. Comparators: When available, control groups were included for between-group comparisons using meta-analysis.
4. Outcomes: Studies assessing changes in PA-related outcomes (eg, steps) or SB were included. These outcomes were required to be continuous data, obtained either through device-based measurements or a 6-minute walking test or subjectively via self-reported questionnaires.
5. Study designs: Only RCTs were eligible.
6. Publication status: Full-text research papers were eligible, while conference proceedings, dissertations, and grey literature were excluded.
7. Language: Only English-language studies were included due to the researchers’ language proficiency.

### Information Sources

As the term “gamification” gained widespread adoption in 2010 [13], we set this year as the starting point of our search. We systematically searched 7 electronic databases—Ovid MEDLINE, PubMed, Web of Science, Embase, Scopus, Cochrane, and CINAHL—for studies published between January 1, 2010, and February 3, 2024. To ensure comprehensive coverage, we manually screened reference lists and studies included in relevant systematic reviews and meta-analyses for additional eligible studies.

### Search Strategy

The search strategy, informed by previous systematic reviews [19], targeted 6 key topics: CVD, gamification, mHealth, PA, SB, and RCTs. It was initially formulated for Ovid MEDLINE and subsequently adapted for use in the other databases.

### Selection Process

The search results were exported into EndNote (version 20.6) for document management, and duplicates were removed both automatically and manually. In total, 2 independent reviewers (Tianzhuo Y and Tianyue Y) screened the retrieved records by title, abstract, and full text to identify potentially relevant studies. A third reviewer (FL) resolved any disagreements between the reviewers.

### Data Extraction

In total, 2 independent reviewers (Tianzhuo Y and Tianyue Y) extracted and verified the data, with FL arbitrating disagreements. Data were organized into preestablished Microsoft Excel sheets, capturing study characteristics (author, year, and country), participant details (sample size, age, sex, ethnicity, and diagnosis), and outcomes (measurement tools, methods, units, data at different time points). The intervention details were described using the Template for Intervention Description and Replication (TIDieR) checklist [29], covering aspects such as Why (theoretical framework), What (materials, procedures, and game design elements of gamification intervention), Who provided (intervention provider), How (mode of delivery), Where (location), When and How much (duration and frequency), Tailoring (eg, individualized goal setting), Modifications, and How well (adherence and attrition). Game design elements were categorized based on the gamification persuasion architecture and its 7 persuasion strategies [24]. Additionally, the checklist was used to assess the reporting completeness of each intervention, with items rated as “present,” “absent,” or “unclear.”

### Risk of Bias

The risk of bias was assessed using the Revised Cochrane risk-of-bias tool for randomized trials (RoB 2), which evaluates 5 aspects: randomization process, deviations from intended interventions, missing outcome data, measurement of the outcomes, and selection of the reported results [30]. In total, 2 reviewers (Tianzhuo Y and Tianyue Y) independently performed the assessments, and any discrepancies were resolved through discussion with a third reviewer (FL).

### Effect Measures

Studies reporting PA data as mean (SD) or mean differences with SD of the differences were eligible for inclusion in the meta-analysis. Data reported as medians and interquartile ranges were also included after transformation into means and SD [31].

### Synthesis Methods

Characteristics and TIDieR findings were synthesized narratively, with frequencies and percentages summarized in tables. This qualitative review included all studies meeting the eligibility criteria, even if data could not be obtained for quantitative analysis. Statistical analyses were conducted using R (version 4.3.2). An overall meta-analysis was performed to determine the summary effect, with additional meta-analyses for follow-up effects and specific outcomes (eg, daily steps) when sufficient data were available. The standardized mean difference was applied to calculate effect sizes for continuous variables measured with different instruments, while the mean difference was used for those measured with similar instruments. To address small-sample bias in the included studies, we calculated the effect size Hedges *g* [32], which adjusts Cohen *d* for small-sample bias. A Hedges g of 0.20 indicates a small effect, 0.50 a moderate effect, and 0.80 a large effect [33]. Given the expected between-study heterogeneity, a random-effects model was used to pool effect sizes. To mitigate potential biases associated with the DerSimonian-Laird method, particularly in cases of few studies and high heterogeneity [34], the Sidik-Jonkman method with Knapp-Hartung adjustments was used for more robust variance estimates [35]. According to the Cochrane Handbook [36], when a study included multiple intervention groups and aimed to compare the effects of different interventions, each intervention group was treated as a separate study. The “shared” control group was split into 2 or more groups with reduced sample sizes for comparison. Only the total number of participants in the control group was divided for continuous outcomes, while the mean and SD remained unchanged.

### Statistical Heterogeneity

To investigate sources of heterogeneity, we first identified potential outliers, defined as studies with effect sizes that were extreme and significantly deviated from the overall effect [37]. An influence analysis was then performed using the leave-one-out method to determine which studies exerted the greatest impact on the pooled estimate and to assess whether this influence distorted the overall results [38]. Additionally, we used a Baujat plot, a diagnostic tool designed to identify studies that contribute disproportionately to heterogeneity in a meta-analysis [39]. Finally, a sensitivity analysis was conducted to evaluate the robustness of the findings by excluding studies with a high risk of bias, significant heterogeneity, or identified outliers.

The *I^2^* statistic reflects the proportion of variance in observed effects attributable to variance in true effects but does not indicate the extent to which effect sizes vary across studies. Consequently, categorizing heterogeneity as low, moderate, or high based solely on the *I^2^* statistic is not recommended [40]. In this meta-analysis, PIs were used to quantify heterogeneity [38]. PIs represent the range within which the effect size of a new study, randomly chosen from the same population as those included in the meta-analysis, is likely to fall [41]. Unlike CI, PIs provide valuable clinical decision-making insights by estimating the likely intervention effect in future studies [25]. PIs also use the same scale as the effect size, illustrating both the interval’s width and limits, which helps determine whether the intervention consistently produces beneficial effects or has the potential for harm. PIs can be calculated when a meta-analysis includes at least 3 studies [38] and are most suitable when the included studies have a low risk of bias [36]. Therefore, we calculated 95% PIs for each meta-analysis.

### Meta-Regression

To evaluate the most effective game design elements, we performed a meta-regression using a multimodel inference approach. Game design elements were treated as predictors to identify the best combination and the most influential predictor overall [42]. We used the Sidik-Jonkman random-effect model to pool effect sizes and the Knapp-Hartung adjustment method to calculate the test statistic and CI. The small sample-correction Akaike’s information criterion (AICc) was applied as the evaluation criterion for the fitted model.

### Certainty Assessment

We assessed the quality of the evidence using the web-based version GRADEpro GDT (Grading of Recommendations Assessment, Development and Evaluation professional guideline development tool). Although RCT evidence typically starts at a high-quality rating, we evaluated the following domains to determine any necessary downgrades: (1) risk of bias, (2) inconsistency, (3) indirectness, (4) imprecision, and (5) publication bias [43].

### Protocol Deviations

We made the following protocol deviations to facilitate a more comprehensive synthesis of gamification interventions for individuals with CVD. First, the TIDieR checklist was used for narrative synthesis. Second, the Revised Cochrane risk-of-bias tool was used for bias assessment. Third, effect sizes were calculated using Hedges *g*, with CI adjusted via the Sidik-Jonkman method and the Knapp-Hartung adjustment. Fourth, all analyses were conducted using R (version 4.3.2). Last, outlier detection and influential analysis were performed to explore sources of heterogeneity instead of subgroup analyses, and 95% PIs were plotted to measure interstudy heterogeneity in the meta-analysis. No additional protocol deviations from the PROSPERO (International Prospective Register of Systematic Reviews) registration were made.

## Results

### Study Selection

**Figure 1:**
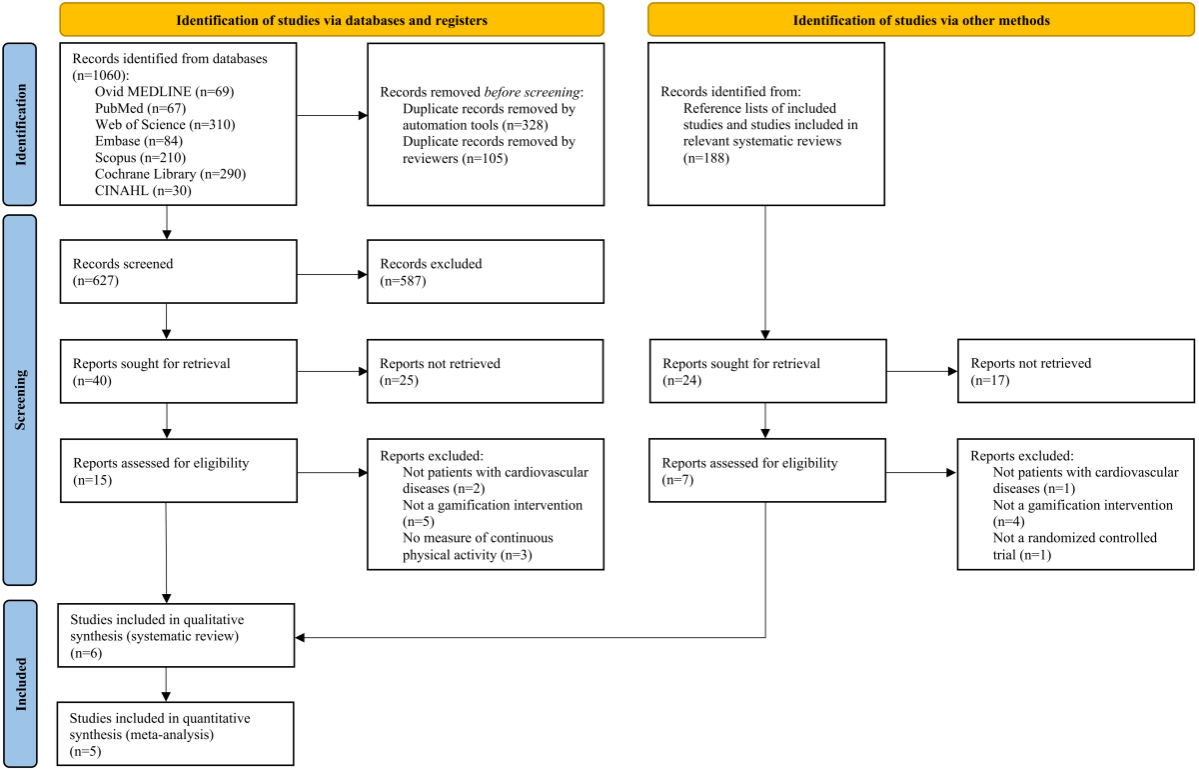
Flowchart for study identification, screening, eligibility, and inclusion.

The initial search yielded 1060 records. The detailed search strategies for each database are provided in Table S1 in Multimedia Appendix 1. Additionally, 188 records were identified through manual searches of reference lists and studies included in relevant systematic reviews. After removing duplicates and screening titles and abstracts, 22 full-text papers were assessed for eligibility. In total, 6 studies met the inclusion criteria and were included in the narrative synthesis. Among them, 5 studies were eligible for inclusion in the meta-analysis. The study selection process is illustrated in Figure 1.

### Study Characteristics

#### Overview

Table S2 in Multimedia Appendix 1 summarizes the characteristics of the included studies. All studies were published within the past 3 years. The majority were conducted in the United States (n=3, 50%), with the remainder conducted in China, Australia, and Germany. A total of 1109 participants were included across the studies, with an average age of 59 years or older and sample sizes ranging from 34 [21] to 500 [44]. Males comprised 55.3% (n=613) of the total sample. Participants were primarily diagnosed with coronary heart disease, heart failure, stroke, atherosclerotic CVD, or PAD.

#### Intervention Characteristics According to the TIDieR Checklist

A summary of the intervention characteristics, including “Why,” “How,” “How long,” “Tailoring,” and “How well,” is provided in Table S3 in Multimedia Appendix 1. Details related to “What” are briefly described below and further summarized in Table S2 in Multimedia Appendix 1.

#### Theoretical Framework (Why)

All but one study [17] used a theoretical framework to guide their interventions, with 2 studies incorporating 2 distinct theories [16,44]. The most frequently applied framework was behavioral economics principles (BEPs) (n=3), followed by self-determination theory (SDT) [16], social cognitive theory (SCT) [27], goal-setting theory (GST) [44], and the Fogg behavioral model (FBM) [45].

#### Intervention Content (What)

All included studies incorporated game design elements, ranging from 4 [44] to 7 elements [16,45]. Among the 11 identified elements, “Goals” and “Feedback” were the most frequently used (n=5, 83.3%), while “Social support” and “Collaboration” were the least used (n=1, 16.7%). None of the studies applied all 7 gamification persuasion strategies. Detailed descriptions of the gamification interventions are provided in Table S4 in Multimedia Appendix 1.

In the control groups, participants in 3 studies were asked only to use wearable devices for passive PA monitoring without receiving additional interventions [21,44,45]. One study required control group participants to use the same application as the intervention group **but without the active engagement of the game design elements** [16]. In 2 studies, control group participants received standard care only [17,27].

#### Mode of Delivery (How)

Applications were the most commonly used delivery method, with 4 studies using independently designed and developed applications to deliver gamification interventions [16,17,27,45]. In total, 2 studies used the “Way to Health” research technology platform [21,44]. Additionally, 3 studies provided participants with PA feedback via SMS text messages and emails [21,27,44]. Wearable devices used in the 3 studies not only measured PA but also synchronized the data to applications and websites [21,44,45].

#### Intervention and Follow-Up Duration (When and How Much)

The intervention periods ranged from 2 months [21] to 6 months [27]. More than half of the interventions (n=4) were considered medium in duration (≥3 months), one was short (<3 months), and one was long (≥6 months). In total, 2 studies included predefined follow-up periods ranging from 2 months [44] to 3 months [16].

#### Adherence and Attrition (How Well)

Attrition rates varied among the studies: 3 reported low dropout rates (<13%) [16,21,44], 2 had medium rates (13%-26%) [17,45], and 1 reported a high dropout rate (>26%) [27]. Intervention adherence was evaluated through task completion [27] and application usage [17,45].

#### TIDieR Coding

The completeness of reporting across TIDieR items varied, ranging from 41.7% (n=5) [44] to 83.3% (n=10) [45], with an average of 7 out of 12 items adequately reported. The most consistently reported items were brief name (item 1), rationale or theory (item 2), intervention content (item 4), mode of delivery (item 6), and duration of intervention and follow-up (item 8), all covered in all studies. In total, 4 studies (66.7%) tailored participants’ weekly PA goals based on baseline data (item 9) [16,17,21,45]. Only one study reported intervention modifications (item 10) [22]. Similarly, just one study explicitly stated that the intervention was self-delivered by participants (item 5) [27], and another specified that the intervention occurred at the participant’s home or convenient locations (item 7) [45]. Other studies’ descriptions of both items lack clarity. However, since all interventions involve self-managed PA, participants and their homes or a convenient location were expected to serve as intervention providers and locations in other studies. For intervention materials (item 3), 2 of the 3 studies provided descriptions, but the level of detail was insufficient for replication [17,27]. Adherence or fidelity (item 11 and item 12) was reported in 3 studies (50%) [17,27,45]. A detailed summary of TIDieR items’ completeness is presented in Table S5 in Multimedia Appendix 1.

### Outcomes Characteristics

In total, 4 studies focused on steps [16,21,44,45], while others measured moderate-to-vigorous PA [44], quantity in metabolic equivalent of task [27], and walking test distance [17]. Except for one study [27], which used the self-reported Global Physical Activity Questionnaire to assess total PA, all other studies used objective measurement tools, including wearable devices (n=3) [21,44,45], smartphone accelerometers [16], or the 6-minute walking test [17]. A detailed summary of PA-related outcomes is presented in Table S6 in Multimedia Appendix 1.

### Risk of Bias

Among the 6 included studies, 5 had a low overall risk of bias, and one raised some concerns [17] due to not using appropriate analyses to estimate intervention effects (see Figure S1 in Multimedia Appendix 1). All studies were at low risk of bias in domains of the randomization process, missing outcome data, outcome measurement, and selection of the reported results. Detailed risk of bias assessments are provided in Table S7 in Multimedia Appendix 1.

### Results of Syntheses

#### Summary Effect

**Figure 2:**
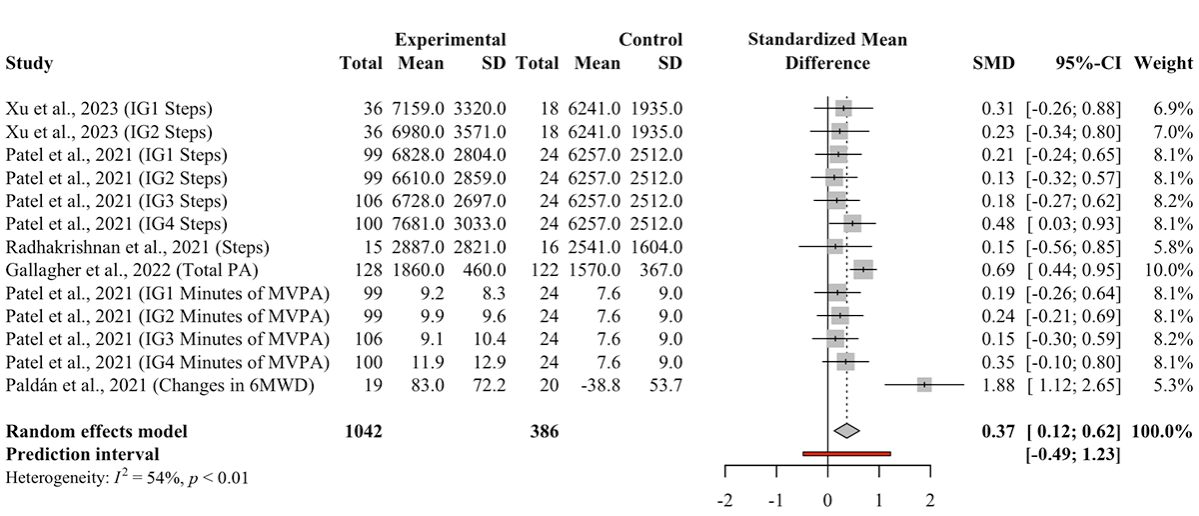
Forest plot for the effect of gamification on PA at postintervention. 6MWD: 6-minute walking distance; IG: intervention group; PA: physical activity; MVPA: moderate-to-vigorous intensity physical activity; SMD: standardized mean difference.

At postintervention, the overall effect size for PA-related outcomes was Hedges *g* of 0.37 (95% CI 0.12-0.62), indicating a statistically significant small-to-moderate effect (Figure 2). Assuming a normal distribution of effects, the PI ranged from –0.49 to 1.23, suggesting that the true effect size for any single population would usually fall within this range. Based on the formula [26], the estimated probability that the true effect of gamification compared to nongamification on PA in individuals with CVD exceeds zero in the new study is 82.1%.

#### Predefined Follow-Up

**Figure 3:**
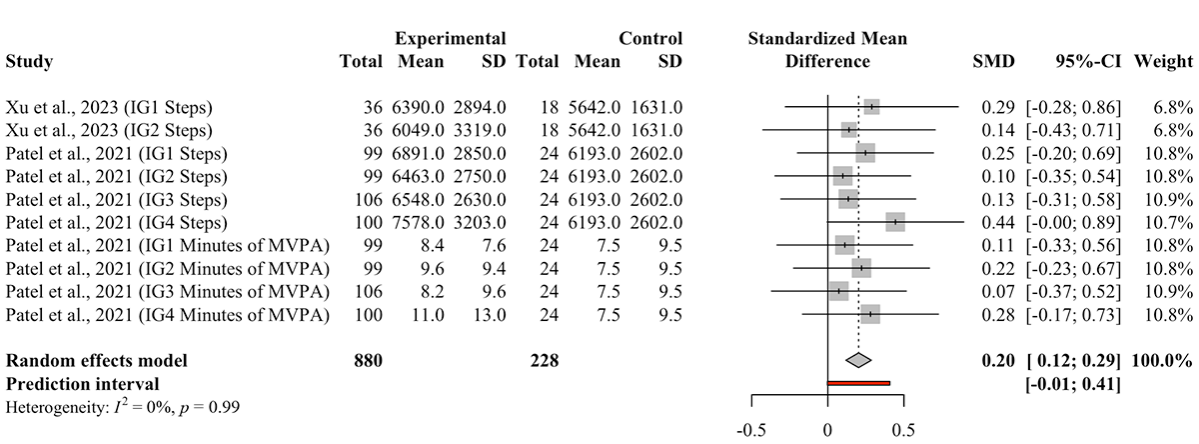
Forest plot for the effect of gamification on PA at the end of the follow-up period. IG: intervention group; PA: physical activity; MVPA: moderate-to-vigorous intensity physical activity; SMD: standardized mean difference.

At the end of the predefined follow-up, gamification interventions resulted in a statistically significant small increase in PA (Hedges *g*=0.20, 95% CI 0.12-0.29; Figure 3). The 95% PI ranged from –0.01 to 0.41, indicating that in approximately 95% of similar studies, the actual effect size for PA would usually fall within this range. Based on the formula [26], the probability that the true effect of a gamification intervention compared to a nongamification intervention on PA exceeds zero in the new study is estimated at 99.7%.

#### Daily Steps

**Figure 4:**
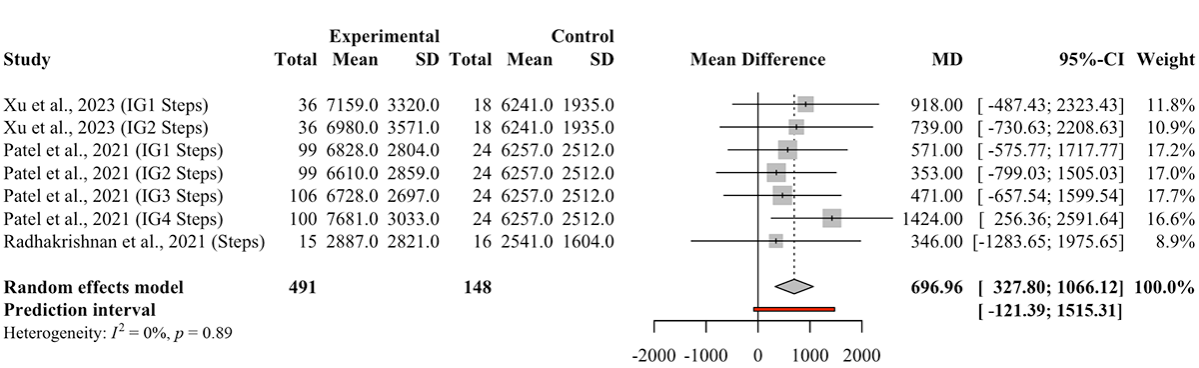
Forest plot for the effect of gamification on daily steps. IG: intervention group; MD: mean difference.

A statistically significant increase in daily steps was observed, with a mean difference of 696.96 daily steps (Figure 4). The 95% PI indicated that the change in daily steps for participants using gamification ranged from a decrease of 121.39 steps to an increase of 1515.31 steps. Based on the formula [26], the probability that the true effect of gamification will lead to an increase in daily steps compared to nongamification in the new study is estimated at 80.9%.

### Outliers and Influence Analyses

In the short-term PA analysis, substantial statistical heterogeneity was observed. To this, we first identified outliers, with one study by Paldán et al [17] flagged as an outlier. Leave-one-out analyses revealed that sequential removal of each study did not significantly affect the overall effect size, which ranged from a Hedges *g* of 0.32 (95% CI 0.19-0.45) to 0.39 (95% CI 0.12-0.66; see Figure S2 in Multimedia Appendix 1). The Baujat plot also identified the study by Paldán et al [17] as contributing most to the heterogeneity (influence value is 14.61; see Figure S3 in Multimedia Appendix 1). Given its outlier status and concerns about the risk of bias, we excluded this study from the sensitivity analysis. After exclusion, we still obtained a statistically significant effect of a Hedges g of 0.32 (95% CI 0.19-0.45), representing a small effect with minor heterogeneity (95% PI 0.02-0.62; see Figure S4 in Multimedia Appendix 1).

### Meta-Regression

Meta-regression using the multimodel inference method identified the top 5 models, ranked by increasing AICc values, with “Feedback plus Goals” having the lowest AICc (4.0) and thus showing the best fit. However, other predictor combinations had similar AICc values, making it difficult to determine a definitive “best” model. Notably, all top 5 models included the predictor “Feedback,” suggesting it may be particularly important. The top 5 models are summarized in Table S8 in Multimedia Appendix 1. As shown in Figure S5 in Multimedia Appendix 1, only 10 of the 11 game design elements were included in the predictor importance plot, as none of the studies in the meta-analysis used the element “Social support.” The plot illustrates the average importance of each predictor across all models, highlighting “Feedback” as the most important predictor (importance value is 0.71), followed by “Avatars” (importance value is 0.59).

### Certainty Assessment

After conducting the sensitivity analysis, the quality of evidence for short-term PA, predefined follow-up PA, and daily steps was rated as high (see Table S9 in Multimedia Appendix 1).

## Discussion

### Summary Effect

In total, 6 studies were included in this review, all published within the past 3 years, reflecting the growing interest in gamification interventions in the field of CVD. This meta-analysis of 1075 participants from 5 RCTs demonstrated that mHealth-based gamification interventions had a statistically significant effect on overall PA over a mean intervention duration of 3.8 months (Hedges *g*=0.32, 95% CI 0.19-0.45 after sensitivity analysis), indicating that gamification interventions effectively promote PA in individuals with CVD. This effect remained robust even after conducting different influence analyses, ie, the leave-one-out method.

These findings align with those of Mazeas et al [19], who assessed the impact of gamification interventions on PA in a broader population. Their study, which included healthy individuals and those with chronic diseases, demonstrated that a 12-week gamification intervention statistically significantly improved PA levels (Hedges *g*=0.42, 95% CI 0.14-0.69 after sensitivity analysis). However, the mean age of participants in Mazeas et al’s study was 35.7 years [19], raising questions about the generalizability of results to older individuals [46]. In contrast, the present meta-analysis focuses on participants with a mean age exceeding 59 years, addressing, to some extent, the research gap regarding the effects of gamification interventions on PA in older individuals. Nonetheless, future research is needed to comprehensively evaluate the impact of gamification interventions on older adults [47].

### Follow-Up Effect

When analyzing the predefined follow-up effects of gamification interventions on PA, we observed a statistically significant effect size (Hedges *g*=0.20, 95% CI 0.12-0.29) at a mean follow-up time of 2.5 months after the intervention ended. These findings suggest that the effects of gamification persist beyond the intervention period, although the maintenance effect diminishes over time. Similarly, Mazeas et al [19] reported a statistically significant effect at a longer follow-up period (mean 3.6 months) with a weaker effect size (Hedges *g*=0.15, 95% CI 0.07-0.23 after sensitivity analysis). This consistency between studies indicates that gamification is not merely a short-term novelty effect for increasing PA. However, additional research is warranted to explore the longer-term sustainability of gamification interventions, particularly beyond 6 months.

### Effect on Daily Steps

The impact of the gamification intervention on daily steps was similarly promising. The meta-analysis demonstrated a statistically significant effect of mHealth-based gamification interventions, with participants in the intervention group increasing their daily steps by 696.96 compared to the control group (Hedges *g*=696.96, 95% CI 327.80-1066.12). In contrast, Mazeas et al [19] reported a larger increase of 1609.56 steps per day among participants in their gamification group. This discrepancy could be attributed to differences in participant characteristics; this review exclusively included individuals with CVD, who are generally less physically active than those without CVD [48]. Nevertheless, individuals with CVD often derive greater health benefits from the same level of PA [49], which underscores the potential meaning of our findings.

Furthermore, while current guidelines emphasize the intensity of PA [5], Oja et al [50] concluded that even moderate walking intervention yields cardiovascular health benefits. Paluch et al [51] demonstrated that higher daily steps were associated with a progressively lower risk of CVD among older adults. Banach et al [52] found that an increase of just 500 steps per day was linked to a 7% reduction in cardiovascular mortality. Based on the PI, in a new study, the probability that gamification interventions would increase daily steps by at least 500 in individuals with CVD is estimated to be 59.8%. This highlights the potential of gamification interventions to statistically and clinically reduce CVD risk by promoting higher daily steps.

### Statistical Heterogeneity

The meta-analyses conducted in this review all demonstrated statistically significant effects, with 95% CI consistently located on the same side of the null. However, due to between-study heterogeneity, the corresponding 95% PI spanned both sides of the null. While most PIs were primarily to the right of the null, indicating that gamification interventions are generally effective, the overlap with the null suggests that their effectiveness may vary in certain contexts. The uncertainty reflected by the PI is tied to how closely future studies align with the characteristics of the completed studies [26]. This indicates that optimizing gamification interventions based on existing studies may help minimize null results in future research.

### Theoretical Foundations of Gamification

#### Overview

A growing body of research has examined how various theoretical foundations can inform the design and effectiveness of gamification interventions [53]. A systematic review of mHealth-based gamification interventions for promoting PA participation found that interventions with theoretical guidance were more effective than those without [54]. Drawing on the theories applied in the included studies, we discuss how different theoretical foundations, each with distinct focuses, can elucidate the underlying mechanisms of gamification. Currently, the theoretical foundations guiding the development, implementation, and evaluation of gamification interventions can be broadly categorized into 3 main groups [55].

#### Focus on Affect and Motivation

The first category of theoretical foundations emphasizes affect and motivation. SDT is a central representative of this category, evolving over decades into an organic and dialectical meta-theory of human motivation [56]. As the ubiquitous theoretical framework in gamification, SDT has been extensively applied to guide intervention design [55]. For example, Xu et al used game design elements that support SDT’s 3 basic psychological needs—autonomy, competence, and relatedness—to develop a gamification intervention [16]. Satisfying these needs fosters participants’ autonomous motivation, enhancing their sustained participation in PA [57].

GST also belongs to this category, primarily serving to refine and optimize gamification interventions [55]. Patel et al [44] integrated gamification interventions with various goal-setting methods grounded in GST. Their findings revealed that behavioral design gamification interventions led to increased PA, with more significant improvements observed when interventions included self-chosen and immediate goals.

#### Focus on Behavior

The second category of theoretical foundations emphasizes behavior. An example is the FBM, which Radhakrishnan et al used to guide interventions and select digital game elements [45]. FBM is built on 3 core concepts: motivation, ability, and triggers. According to FBM, individuals can perform the target healthy behavior only when they have sufficient motivation, the ability to perform the behavior, and are prompted by appropriate triggers [58].

#### Focus on Learning

The third category of theoretical foundation emphasizes learning and is primarily derived from social psychology, notably SCT [55]. Within the SCT framework, self-efficacy—an individual’s belief in their ability to perform a specific behavior—is regarded as a core determinant of task-oriented behavior [59]. An example of incorporating SCT into gamification is the study by Gallagher et al [27], which used strategies designed to enhance participants’ self-efficacy, such as providing incremental challenges, monitoring and tracking activity performance, and a “coin” reward system.

#### Behavioral Economics Principles

In addition to the 3 main theoretical foundations of gamification, BEP emerged as the most frequently applied theory in the included studies [16,21,44]. Principles such as the “Fresh start effect,” “Prospect theory/Loss aversion,” and “Goal gradient” were ingeniously integrated with game design elements to establish effective “gamification rules” to drive behavior change. A detailed summary of the application and implications of BEP in the included studies is provided in Table S10 in Multimedia Appendix 1. BEP has gained increasing traction in the primary and secondary prevention of CVD. By enabling more targeted and strategic application of these principles, behavioral economics has the potential to serve as a powerful tool for promoting positive clinical outcomes in individuals with CVD [60].

### Game Design Elements

#### Overview

The most frequently used game design elements in the included studies were “Goals” and “Feedback,” followed by “Rewards” and “Progress bars.” These findings align with previous reviews. For example, Davis et al [22] summarized the gamification strategies included in health mobile applications for older adults at high risk for CVD, reporting universal use of “Goal setting” (n=7, 100%) and frequent use of “Rewards (virtual and tangible)” and “Track/show progress” (n=6, 85.7%). Similarly, Xu et al [54] reviewed gamification interventions targeting PA participation and identified “Goal Setting” as the most commonly used game design element (n=30, 60%), followed by “Progress bars” (n=26, 52%), “Rewards” (n=25, 50%), and “Feedback” (n=21, 42%). These findings suggest that goal setting, performance feedback, progress visualization, and rewards may be among the most appealing components of gamification interventions.

Using meta-regression multimodel inference, we identified “Feedback” as the most important predictor, followed by “Avatars.” While multimodel inference helps to provide a comprehensive overview of predictors influencing effect sizes, it remains an exploratory method. Moreover, the small number of included studies limits the generalizability of these findings. To offer a broader perspective, we discussed all 11 game design elements included in the systematic review, providing insights and references for designing future gamification interventions.

#### Goals

Setting goals facilitates behavioral change by focusing attention and effort while enhancing perseverance toward achieving specific proficiency levels [61,62]. Most studies in this review implemented “gradual goals” that were updated weekly [16,17,44]. Evidence suggests that adjusting goals weekly or bi-weekly, when necessary, better supports sustained PA behavior change [63]. Combining goal-setting attributes such as feedback, rewards, and task strategies appears to be beneficial in maximizing the effectiveness of interventions on PA behaviors [63]. This validates why goals, feedback, and rewards are among the most frequently used game design elements.

#### Challenges

Paul et al [64] propose that layered or incremental challenges are among the most effective gamification strategies, particularly when progress can be tracked through wearable devices. For example, the “MyHeartMate” application used incremental challenges but lacked an objective method to track their completion [27]. Challenges can also serve as an effective way to drive other gamification mechanisms [65]. For instance, challenges can be nested within badges, as demonstrated in the “TrackPAD” application, where completing a 7-consecutive-day training challenge unlocks the corresponding badge [17]. Similarly, challenges can be linked to a points system, as seen in the Samsung Health application, where participants earn points for completing challenges and progress to higher levels [66]. Through ongoing challenges, participants may be motivated to continue using an application, especially when these challenges validate their understanding of its goals [65]. Miller et al [67] emphasize the importance of regularly updating challenges to ensure participants have sufficient choices, which helps maintain their motivation to use the application.

#### Points and Levels

“Points” play an essential place in gamification environments [68]. They serve as a unit of measurement for game scores and can be assigned metaphorical icons or titles, commonly called “Levels,” to represent participants’ progress [67]. Points are also considered a form of gamification reward [69]. Using points and levels can incentivize participants to consistently engage with mHealth-based gamification interventions for PA and to acquire knowledge about relevant CVD. Additionally, points can serve as a feedback mechanism; for instance, they can stay or be deducted to indicate whether a step goal for the day has been achieved [16,21,44]. By accumulating points and progressing through point-based levels, participants may experience an enhanced sense of competence [70,71].

#### Feedback

“Feedback” is a crucial component of remotely delivered PA interventions [72], and PA interventions that incorporate performance feedback tend to achieve greater success [73]. Our meta-regression results identified “Feedback” as the most important predictor, reinforcing this perspective. Zuckerman and Gal-Oz [74] developed the “StepByStep” application to encourage participants to walk more. However, participants reported a lack of statistics or graph representations of their daily steps progress and suggested including their personal activity history to facilitate postactivity reflection. This underscores the value of summarized feedback. Moreover, there is a correlation between the amount of PA and the frequency of receiving summary feedback. Research has shown that participants who received daily feedback demonstrated more significant increases in daily steps than those receiving weekly feedback [72].

#### Rewards

Gamification focuses on motivating participants, often by leveraging extrinsic and intrinsic motivation [75]. While extrinsic motivation may drive short-term behavioral changes, intrinsic motivation is more strongly linked to sustained behavior changes over time [76,77]. Rewards in gamification are thought to stimulate intrinsic motivation by fulfilling the basic psychological needs outlined in SDT [78]. Ideally, rewards should primarily promote autonomy and competence while providing enjoyment and fun without being perceived as overly controlling [79]. However, practical research indicates that not all types of rewards effectively maintain or enhance intrinsic motivation. Lewis et al recommend prioritizing verbal, task-noncontingent, and glory rewards while modifying tangible and task-contingent rewards to reduce the perception of control and foster intrinsic motivation [69]. Future research should focus on identifying specific rewards that evoke feelings of volition, willingness, and enjoyment while avoiding those associated with tension, unwillingness, or coercion [80].

#### Progress Bars

“Feedback” provides daily or weekly summaries of PA, while “Progress bars” offer real-time feedback. Health gamification operates at the intersection of persuasive technologies, serious games, and personal informatics. Like personal informatics, gamification often centers on tracking individual behaviors [15], aligning with the quantified self-movement concept [81]. Using accelerometers built into wearable devices or mobile phones to track participants’ PA and visualize the progress through progress bars represents a straightforward yet quantifiable system that can effectively prompt behavior change [74]. One possible reason for its effectiveness is its ability to support reflection in action by delivering real-time feedback (ie, “Progress bars”) and reflection after action by summarizing prior activities (ie, “Feedback”). Both modes of reflection can potentially motivate participants to change their current level of PA [82].

#### Leaderboards

“Progress bars” monitor individual progress, while “Leaderboards” enable social comparison. Half of the studies in this review used “Leaderboards,” highlighting their popularity and effectiveness as a gamification strategy [83]. According to Zichermann and Cunningham [65], applications should offer switchable leaderboards, allowing users to compare rankings within their social network or against a global user base, offering a broader context to measure their progress. Additional columns and filters can be incorporated to provide other relevant elements aligned with the application’s design and purpose. For instance, the “TrackPAD” application implemented 4 different types of leaderboards (ie, number of steps in single training sessions, number of completed training sessions, total minutes of PA, and percent increase of PA) [17]. Leaderboards that facilitate social comparison help participants assess their performance relative to others. The dynamic comparison process can elevate individuals’ expectations for goal achievement, ultimately leading to increased PA levels [84].

#### Social Support

Social support is critical in sustaining engagement with health behavior change interventions and enhancing outcomes [85]. Among the included studies, social support was often facilitated through participants’ existing social networks, involving significant others such as spouses, friends, or family members. These individuals provided emotional support, positively influenced participants’ health behaviors [21]. Research indicates that higher levels of social support, especially from family, are associated with increased PA levels in older adults [86]. However, future studies should prioritize assessing participants’ levels of social engagement and exploring strategies to enhance social support for populations at high risk of social isolation, such as older adults [87]. An RCT by Greysen et al [87] investigated the impact of gamification combined with a social support partner intervention on daily steps among hospitalized adults after discharge. The study found that gamification-based social incentives improved mobility only in participants with higher levels of social engagement.

#### Collaboration and Competition

One study found that adherence rates were 66% higher when participants used a team-based application than exercise alone [85]. Increased adherence rates may be associated with improved health behaviors. However, the only study in this review that incorporated a “Collaborative” game design element reported “poor” results: while there was a statistically significant increase in mean daily steps in the individual group compared to the control group, no statistically significant difference was observed in the team group [16]. This outcome may be attributed to negative participants reducing their team’s overall motivation and the benefits of social bonding being more fully realized when team members are equally engaged [22]. Additionally, Xu et al [16] suggested that collaboration may not be effective without established social relationships among participants. Supporting this view, a previous RCT demonstrated that gamification collaboration statistically significantly increased daily steps among household members [88], highlighting the potential effectiveness of behavior change programs when participants are engaged together and socially connected [89]. However, collaborating with family members may not always be effective for participants with specific diseases, such as CVD, particularly if family members lack equal motivation to engage in health behavior change. Future research should focus on identifying best practices for organizing effective collaborative teams, considering factors such as team size, member relationships, and incentives to balance peer support, team responsibility reinforcement, and individual achievement rewards.

Patel et al [90] introduced a novel perspective for interventions involving participants without preexisting social relationships. In a 24-week PA intervention with overweight or obese adults unfamiliar with each other, they found that the gamification elements supporting collaboration and competition all statistically significantly increased PA compared to the control group, with competition showing the strongest effect. This suggests that competition may be more effective than collaboration in the absence of prior social relationships. Competing with teammates or other teams stimulates a powerful innate human drive [91]. However, the game design element “Competition” was not used in the included studies, likely due to concerns that excessive PA could be counterproductive for individuals with CVD [16]. Future studies could explore competition among individuals with CVD based on achieving daily PA goals rather than total activity levels. This approach may reduce the risk of overexercising while using the game design element of competition safely and effectively.

#### Avatars

Researchers now widely agree on the central theoretical architecture of gamification, emphasizing intrinsic and extrinsic motivation as defined by SDT [92]. Avatars provide significant visual feedback with endogenous value. Changes in avatars are linked to participants’ PA behaviors. This connection fosters a sense of participation and engagement, which can sustain and enhance motivation and willingness to engage in PA [93]. In our meta-regression, “Avatar” emerged as the second most important predictor, confirming this connection. According to SDT, sociocontextual events (eg, feedback, communication, or rewards) that generate a sense of competence during actions can enhance intrinsic motivation. Several applications demonstrate this principle. For instance, the “MyHeartMate” application uses a cartoon heart avatar to virtually represent the health status of individuals with coronary artery disease [27]. The “Heart Health Mountain” application for individuals with heart failure incorporates communication through avatars [45]. The “STARFISH” application for individuals with stroke translates participants’ active PA into rewards that customize their colorful fish avatars in a virtual fish tank [64]. Future research should focus on designing attractive avatars tailored to specific populations and crafting interactions with avatars to enhance intrinsic motivation for behavior and subsequently improve PA.

### Limitations

This review has 4 main limitations. First, the small number of included studies, some of which were feasibility or pilot studies, resulted in small sample sizes and underpowered analyses. However, this limitation reflects the emerging momentum of gamification interventions as secondary prevention for CVD. Second, all studies incorporated multiple gamification elements, making it challenging to isolate the effects of individual elements. While we conducted meta-regression multimodel inference to explore this, the results remain exploratory. Further research focusing on the independent effects of specific gamification elements is needed. Third, although we had an interest in exploring whether gamification can reduce SB, none of the included studies evaluated that outcome. Designing gamification interventions targeting SB may be a valuable direction for future research. Finally, none of the studies conducted cost-effectiveness analyses, which are crucial for evaluating the public health impact of gamification interventions and guiding resource allocation. Incorporating cost-effectiveness analyses in future studies could provide evidence to support the adoption of more efficient interventions and replace fewer effective ones [94].

### Conclusions

In conclusion, gamification interventions show promise in promoting PA, particularly in increasing daily steps among individuals with CVD. While the effects may diminish over time, their persistence during follow-up suggests that gamification is not merely a novelty effect. However, attributing effects to individual game design elements remains challenging, as no studies have independently tested their impacts. Future studies should include larger sample sizes, longer durations, and more rigorous designs to further explore the effectiveness and persistence of gamification interventions and the impact of individual game design elements.

## Appendix

Multimedia Appendix 1 Related multimedia appendix tables and figures.

Multimedia Appendix 2 PRISMA 2020 Checklist.

## Abbreviations

AICc: sample-correction Akaike’s information criterion
BEP: behavioral economics principles
CVD: cardiovascular disease
FBM: Fogg behavioral model
GST: goal-setting theory
mHealth: mobile health
PA: physical activity
PAD: peripheral artery disease
PI: prediction intervals
PRISMA: Preferred Reporting Items for Systematic Reviews and Meta-Analyses
PROSPERO: International Prospective Register of Systematic Reviews
RCT: randomized controlled trial
SB: sedentary behavior
SCT: social cognitive theory
SDT: self-determination theory
TIDieR: Template for Intervention Description and Replication

## Data Availability

All data generated or analyzed during this study are included in the supplementary files accompanying this published article.
