# Supplementary material for: Effectiveness of Mobile Health–Based Gamification Interventions for Improving Physical Activity in Individuals With Cardiovascular Diseases: Systematic Review and Meta-Analysis of Randomized Controlled Trials
Source: JMIR Serious Games. 2025 Jan 24;13:e64410. doi: 10.2196/64410 (PMC11806271; doi:10.2196/64410)
Supplement: Multimedia Appendix 1 [file games_v13i1e64410_app1.pdf]

# Related Multimedia Appendix Tables and Figures

## Content

Table S1. The search strategy of each database. 1
Table S2. Characteristics of the included studies. 9
Table S3. Intervention characteristics with TIDieR headings. 10
Table S4. Details of the gamification intervention. 11
Table S5. TIDieR reporting in each study. 13
Table S6. Outcomes data of the included studies. 13
Table S7. Details of risk of bias assessment of included studies. 14
Table S8. Best 5 models (model selection table). 15
Table S9. Summary of the quality of the evidence for gamification versus control. 15
Table S10. Summary of the application of the principles of behavioral economics. 15
Figure S1. Risk of bias. 16
Figure S2. Forest plot representing Leave-one-out analyses ordered by effect size (Hedge's g; low to high). 16
Figure S3. Baujat plot representing each study's contribution to the overall heterogeneity (measured by Cochran's Q) as a function of its influence on the pooled effect size. 17
Figure S4. Forest plot for the effect of short-term physical activity after sensitivity analysis. 17
Figure S5. Predictor importance plot of game design elements. 18

Table S1. The search strategy of each database.

| Search terms | | Results |
|---|---|---|
| *Ovid MEDLINE* | | |
| #1 | exp Cardiovascular Diseases/ | 2764151 |
| #2 | exp Heart Diseases/ | 1289263 |
| #3 | (cardiovascular disease$ or CVD or cardiocerebrovascular disease$ or cardiovascular event$ or heart disease$).ab,ti. | 445973 |
| #4 | exp Stroke/ | 178650 |
| #5 | exp Myocardial Infarction/ | 196404 |
| #6 | (stroke or myocardial infarction or MI or heart infarction).ab,ti. | 517341 |
| #7 | exp Myocardial Ischemia/ | 477238 |
| #8 | (isch?emic heart disease$ or IHD or myocardial isch?emia).ab,ti. | 75132 |
| #9 | exp Heart Failure/ | 151582 |
| #10 | (heart failure or cardiac failure).ab,ti. | 224628 |
| #11 | exp Coronary Artery Disease/ | 79003 |
| #12 | (coronary artery disease$ or CAD or coronary heart disease$ or CHD).ab,ti. | 187281 |
| #13 | exp Cerebrovascular Disorders/ | 437030 |
| #14 | exp Cerebral Arterial Diseases/ | 21577 |
| #15 | (cerebrovascular disorder$ or cerebrovascular disease$ or CBVD or cerebral arterial disease$ or cerebral artery disease$).ab,ti. | 27862 |
| #16 | (cerebrovascular accident$ or cerebrovascular attack$ or CVA).ab,ti. | 10420 |
| #17 | exp Cerebral Infarction/ or cerebral infarction.ab,ti. | 46895 |
| #18 | exp Peripheral Vascular Diseases/ | 65504 |
| #19 | exp Peripheral Arterial Disease/ | 19324 |
| #20 | (peripheral vascular disease$ or peripheral arterial disease$ or peripheral artery disease$ or PVD or PAD).ab,ti. | 54176 |
| #21 | or/1-20 | 3169366 |
| #22 | exp gamification/ | 126 |
| #23 | exp exergaming/ | 181 |
| #24 | gamification.ab,ti. | 1281 |
| #25 | (exergaming or exer-gaming).ab,ti. | 579 |
| #26 | or/22-25 | 1953 |
| #27 | (game or games or gamified or gaming or gameful$ or multiplayer or player or players or playing).ab,ti. | 167544 |
| #28 | exp Telemedicine/ | 46533 |
| #29 | (telemedicine or telehealth or e-Health or m-Health or eHealth or mHealth or mobile health or digital health).ab,ti. | 50366 |
| #30 | exp Mobile Applications/ | 12166 |
| #31 | (mobile application$ or application$ or app or apps).ab,ti. | 1706694 |
| #32 | exp Internet/ | 99502 |
| #33 | (internet or online or mobile or web based or web-based).ab,ti. | 448766 |
| #34 | exp Smartphone/ | 9674 |
| #35 | (smartphone$ or smart phone$ or phone$, smart).ab,ti. | 25614 |
| #36 | exp Cell Phone/ | 23208 |
| #37 | (cell phone$ or cellphone$ or mobile phone$).ab,ti. | 15476 |
| #38 | (iPhone or android or iOS).ab,ti. | 6936 |
| #39 | exp Wearable Electronic Devices/ or wearable electronic device$.ab,ti. | 20200 |
| #40 | exp Digital Technology/ or digital technolog$.ab,ti. | 5730 |
| #41 | (website$ or digital$ or system$ or electronic$ or technolog$ or device$ or framework$).ab,ti. | 6171253 |
| #42 | Social Support/ | 79541 |
| #43 | exp Social Media/ or social media.ab,ti. | 36211 |
| #44 | Social Networking/ | 5570 |
| #45 | (facebook or network$, social or social network$).ab,ti. | 31033 |
| #46 | or/28-45 | 7615205 |
| #47 | 27 and 46 | 56723 |
| #48 | 26 or 47 | 57896 |
| #49 | randomized controlled trial.pt. | 608097 |
| #50 | controlled clinical trial.pt. | 95549 |
| #51 | randomi#ed.ab. | 756248 |
| #52 | placebo.ab. | 245439 |
| #53 | clinical trials as topic.sh. | 201721 |

Table S1. Continued.

| Search terms | | Results |
|---|---|---|
| #54 | randomly.ab. | 426352 |
| #55 | trial.ti. | 302188 |
| #56 | or/49-55 | 1625312 |
| #57 | exp animals/ not humans.sh. | 5193699 |
| #58 | 56 not 57 | 1498908 |
| #59 | exp Exercise/ | 253089 |
| #60 | (exercise or exercises or active living or active transport or activities, physical or activity, physical or physical activities or physical activity).ab,ti. | 478896 |
| #61 | exp Exercise Therapy/ or exercise therapy.ab,ti. | 66892 |
| #62 | exp Physical Fitness/ or physical fitness.ab,ti. | 42399 |
| #63 | fitness.ab,ti. | 98027 |
| #64 | exp Sedentary Behavior/ or sedentary behavio?r$.ab,ti. | 18536 |
| #65 | (sedent$ or sitting time or inactive or inactivity or walk$).ab,ti. | 310104 |
| #66 | or/59-65 | 877045 |
| #67 | 21 and 48 and 58 and 66 | 135 |
| #68 | limit 59 to (humans and yr="2010 -Current" and "all adult (19 plus years)" and english) | 69 |
| *PubMed* | | |
| #1 | "cardiovascular diseases"[mh] OR "heart diseases"[mh] OR "cardiovascular disease*"[tiab] OR "CVD"[tiab] OR "cardiocerebrovascular disease*"[tiab] OR "cardiovascular event*"[tiab] OR "heart disease*"[tiab] OR "stroke"[mh] OR "stroke"[tiab] OR "myocardial infarction"[mh] OR "myocardial infarction"[tiab] OR "MI"[tiab] OR "heart infarction"[tiab] OR "myocardial ischemia"[mh] OR "ischaemic heart disease*"[tiab] OR "ischemic heart disease*"[tiab] OR "IHD"[tiab] OR "myocardial ischaemia"[tiab] OR "myocardial ischemia"[tiab] OR "heart failure"[mh] OR "heart failure"[tiab] OR "cardiac failure"[tiab] OR "coronary artery disease"[mh] OR "coronary artery disease*"[tiab] OR "CAD"[tiab] OR "coronary heart disease*"[tiab] OR "CHD"[tiab] OR "cerebral arterial diseases"[mh] OR "cerebrovascular disorders"[mh] OR "cerebrovascular disorder*"[tiab] OR "cerebrovascular disease*"[tiab] OR "CBVD"[tiab] OR "cerebral infarction"[mh] OR "cerebrovascular accident*"[tiab] OR "cerebrovascular attack*"[tiab] OR "CVA"[tiab] OR "cerebral arterial disease*"[tiab] OR "cerebral artery disease*"[tiab] OR "peripheral vascular diseases"[mh] OR "peripheral arterial disease"[mh] OR "peripheral vascular disease*"[tiab] OR "peripheral arterial disease*"[tiab] OR "peripheral artery disease*"[tiab] OR "PVD"[tiab] OR "PAD"[tiab] | 3,183,907 |
| #2 | "gamification"[mh] OR "exergaming"[mh] OR "gamification"[tiab] OR "exergaming"[tiab] OR "exer-gaming"[tiab] | 2,363 |
| #3 | "game"[tiab] OR "games"[tiab] OR "gamified"[tiab] OR "gaming"[tiab] OR "gameful*"[tiab] OR "multiplayer"[tiab] OR "player"[tiab] OR "players"[tiab] OR "playing"[tiab] | 168,766 |
| #4 | "telemedicine"[mh] OR "telemedicine"[tiab] OR "telehealth"[tiab] OR "e-Health"[tiab] OR "m-Health"[tiab] OR "eHealth"[tiab] OR "mHealth"[tiab] OR "mobile health"[tiab] OR "digital health"[tiab] OR "mobile applications"[mh] OR "mobile application*"[tiab] OR "applications"[tiab] OR "application"[tiab] OR "app"[tiab] OR "apps"[tiab] OR "Internet"[mh] OR "online"[tiab] OR "mobile"[tiab] OR "internet"[tiab] OR "web based"[tiab] OR "web-based"[tiab] OR "smartphone"[mh] OR "phone smart"[tiab] OR "smart phones"[tiab] OR "smartphones"[tiab] OR "smart phone"[tiab] OR "phones smart"[tiab] OR "smartphone"[tiab] OR "Cell Phone"[mh] OR "cell phone*"[tiab] OR "cellphone*"[tiab] OR "mobile phone*"[tiab] OR "Iphone"[tiab] OR "android"[tiab] OR "iOS"[tiab] OR "wearable electronic devices"[mh] OR "wearable electronic device"[tiab] OR "digital technology"[mh] OR "digital technolog*"[tiab] OR "website"[tiab] OR "digital*"[tiab] OR "system*"[tiab] OR "electronic*"[tiab] OR "technolog*"[tiab] OR "device"[tiab] OR "framework*"[tiab] OR "social support"[mesh:noexp] OR "social media"[mh] OR "social media"[tiab] OR "facebook"[tiab] OR "social networking"[mesh:noexp] OR "online social networking"[mesh:noexp] OR "network*, social"[tiab] OR "social network*"[tiab] | 7,629,119 |
| #5 | #3 AND #4 | 57,605 |
| #6 | #2 OR #5 | 58,906 |
| #7 | "exercise"[mh] OR "exercise*"[tiab] OR "active living"[tiab] OR "active transport"[tiab] OR "activities, physical"[tiab] OR "activity, physical"[tiab] OR"physical activities"[tiab] OR "physical activity"[tiab] OR "exercise therapy"[mh] OR "exercise therapy"[tiab] OR "physical fitness"[mh] OR "physical fitness"[tiab] OR fitness[tiab] OR "sedentary behavior"[mh] OR sedent*[tiab] OR "sitting time"[tiab] OR inactive[tiab] OR inactivity[tiab] OR walk*[tiab] | 893,707 |
| #8 | "randomized controlled trial"[pt] OR "controlled clinical trial"[pt] OR "randomized"[tiab] OR "randomised"[tiab] OR "placebo"[tiab] OR "clinical trials as topic"[mesh:noexp] OR "randomly"[tiab] OR "trial"[ti] | 1,640,130 |
| #9 | "animals"[mh] NOT "humans"[mh] | 5,190,597 |

Table S1. Continued.

| Search terms | | Results |
|---|---|---|
| #10 | #8 NOT #9 | 1,513,557 |
| #11 | #1 AND #6 AND #7 AND #10 | 136 |
| #12 | Filters: Humans, English, Adult: 19+ years, from 2010 - 2024 | 67 |
| *Web of Science Core Collection* | | |
| #1 | TS=("cardiovascular disease*" OR CVD OR "cardiocerebrovascular disease*" OR "cardiovascular event*" OR "heart disease*" OR stroke OR "myocardial infarction" OR MI OR "heart infarction" OR "isch$emic heart disease*" OR "myocardial isch$emia" OR IHD OR "heart failure" OR "cardiac failure" OR "coronary artery disease*" OR CAD OR "coronary heart disease*" OR CHD OR "cerebral arterial disease*" OR " cerebral artery disease*" OR "cerebrovascular disorder*" OR "cerebrovascular disease*" OR CBVD OR "cerebral infarction" OR "cerebrovascular accident*" OR "cerebrovascular attack*" OR CVA OR "peripheral vascular disease*" OR "peripheral arterial disease*" OR " peripheral artery disease*" OR PVD OR PAD) | 1,910,987 |
| #2 | TS=(gamifi* OR exergam* OR exer-gam*) | 14,410 |
| #3 | TS=(game$ OR gamified OR gaming OR gameful$ OR multiplayer OR player$ OR playing OR play) | 2,794,355 |
| #4 | TS=(telemedicine OR telehealth OR e-Health OR m-Health OR eHealth OR mHealth OR "mobile health" OR "digital health" OR "mobile application$" OR application$ OR app$ OR internet OR online OR mobile OR "web based" OR web-based OR smartphone$ OR "smart phone$" OR "phone$, smart" OR cellphone$ OR "cell phone$" OR "mobile phone$" OR iPhone OR android OR iOS OR "wearable electronic device$" OR "digital technolog*" OR website$ OR digital* OR system* OR electronic* OR technolog* OR device$ OR framework* OR "social support" OR "support, social" OR "social media" OR "social network*" OR "network$, social" OR Facebook) | 22,384,253 |
| #5 | #3 AND #4 | 1,112,392 |
| #6 | #2 OR #5 | 1,118,661 |
| #7 | TS=("randomi?ed controlled trial" OR "controlled clinical trial" OR randomi?ed OR placebo OR "clinical trials as topic" OR randomly OR trial) | 2,812,798 |
| #8 | TS=(animals NOT humans) | 1,063,838 |
| #9 | #7 NOT #8 | 2,720,939 |
| #10 | TS=(exercise* OR "active living" OR "active transport" OR "activities, physical" OR "activity, physical" OR "physical activities" OR "physical activity" OR "exercise therapy" OR "physical fitness" OR fitness OR "sedentary behavior*" OR "sedentary behaviour" OR sedent* OR "sitting time" OR inactive OR inactivity OR walk*) | 1,355,441 |
| #11 | #1 AND #6 AND #9 AND #10 | 582 |
| #12 | #1 AND #6 AND #9 AND #10 and 2024 or 2023 or 2022 or 2021 or 2020 or 2019 or 2018 or 2017 or 2016 or 2015 or 2014 or 2013 or 2012 or 2011 or 2010 (Publication Years) and Article (Document Types) and English (Languages) | 310 |
| *Embase* | | |
| #1 | 'cardiovascular disease'/exp | 5,578,938 |
| #2 | 'heart disease'/exp | 2,481,510 |
| #3 | 'cardiovascular disease*':ab,ti OR 'cvd':ab,ti OR 'cardiocerebrovascular disease*':ab,ti OR 'cardiovascular event*':ab,ti OR 'heart disease*':ab,ti | 659,731 |
| #4 | 'stroke*':ab,ti | 515,546 |
| #5 | 'heart infarction'/exp | 475,676 |
| #6 | 'myocardial infarction':ab,ti OR 'mi':ab,ti OR 'heart infarction*':ab,ti | 371,071 |
| #7 | 'heart muscle ischemia'/exp | 103,244 |
| #8 | 'ischemic heart disease'/exp | 796,888 |
| #9 | 'heart muscle ischemia':ab,ti OR 'ischemic heart disease*':ab,ti OR 'ischaemic heart disease*':ab,ti OR 'ihd':ab,ti OR 'myocardial ischaemia':ab,ti OR 'myocardial ischemia':ab,ti | 108,463 |
| #10 | 'heart failure'/exp | 679,745 |
| #11 | 'heart failure':ab,ti OR 'cardiac failure':ab,ti | 376,697 |
| #12 | 'coronary artery disease'/exp | 421,730 |
| #13 | 'coronary artery disease*':ab,ti OR 'cad':ab,ti OR 'coronary heart disease*':ab,ti OR 'chd':ab,ti | 292,851 |
| #14 | 'cerebrovascular disease'/exp | 940,970 |
| #15 | 'cerebral artery disease'/exp | 14,055 |
| #16 | 'cerebrovascular disorder*':ab,ti OR 'cerebrovascular disease*':ab,ti OR 'cbvd':ab,ti OR 'cerebral arterial disease*':ab,ti OR 'cerebral artery disease*':ab,ti | 40,855 |
| #17 | 'cerebrovascular accident'/exp | 445,012 |
| #18 | 'cerebrovascular accident*':ab,ti OR 'cerebrovascular attack*':ab,ti OR 'cva':ab,ti | 19,366 |
| #19 | 'brain infarction'/exp | 92,995 |

Table S1. Continued.

| Search terms | | Results |
|---|---|---|
| #20 | 'cerebral infarction':ab,ti OR 'brain infarction':ab,ti | 28,833 |
| #21 | 'peripheral vascular disease'/exp | 2,444,183 |
| #22 | 'peripheral arterial disease'/exp | 73,324 |
| #23 | 'peripheral vascular disease*':ab,ti OR 'peripheral arterial disease*':ab,ti OR 'peripheral artery disease*':ab,ti OR 'pvd':ab,ti OR 'pad':ab,ti | 85,340 |
| #24 | #1 OR #2 OR #3 OR #4 OR #5 OR #6 OR #7 OR #8 OR #9 OR #10 OR #11 OR #12 OR #13 OR #14 OR #15 OR #16 OR #17 OR #18 OR #19 OR #20 OR #21 OR #22 OR #23 | 5,857,768 |
| #25 | 'gamification'/exp | 560 |
| #26 | 'exergaming'/exp | 367 |
| #27 | 'gamifi*':ab,ti OR 'exergam*':ab,ti | 2,980 |
| #28 | #25 OR #26 OR #27 | 3,222 |
| #29 | 'gam*':ab,ti OR 'game-based':ab,ti OR 'multiplayer':ab,ti OR 'player*':ab,ti OR 'playing':ab,ti | 532,814 |
| #30 | 'telemedicine'/exp | 74,355 |
| #31 | 'telehealth'/exp | 91,534 |
| #32 | ('telemedicine':ab,ti OR 'telehealth':ab,ti OR 'ehealth':ab,ti OR 'mhealth':ab,ti OR 'e-health':ab,ti OR 'm-health':ab,ti) AND 'mobile health':ab,ti OR 'digital health':ab,ti | 10,265 |
| #33 | 'mobile application'/exp | 27,200 |
| #34 | ('mobile application*':ab,ti OR 'application*':ab,ti) AND 'app':ab,ti OR 'apps':ab,ti | 21,963 |
| #35 | 'internet'/exp | 131,692 |
| #36 | 'online system'/exp | 32,236 |
| #37 | 'internet':ab,ti OR 'online system*':ab,ti OR 'online':ab,ti OR 'mobile':ab,ti OR 'web based':ab,ti OR 'web-based':ab,ti | 626,172 |
| #38 | 'smartphone'/exp | 28,270 |
| #39 | 'smartphone*':ab,ti OR 'smart phone*':ab,ti OR 'phone*, smart':ab,ti | 33,100 |
| #40 | 'mobile phone'/exp | 50,049 |
| #41 | 'mobile phone*':ab,ti OR 'cell phone*':ab,ti OR 'cellphone*':ab,ti | 19,415 |
| #42 | 'iphone':ab,ti OR 'android':ab,ti OR 'ios':ab,ti | 24,226 |
| #43 | 'wearable computer'/exp | 9,779 |
| #44 | 'wearable electronic device*':ab,ti | 426 |
| #45 | 'digital technology'/exp | 5,112 |
| #46 | 'digital technolog*':ab,ti | 5,920 |
| #47 | 'website*':ab,ti OR 'digital*':ab,ti OR 'system*':ab,ti OR 'electronic':ab,ti OR 'technolog*':ab,ti OR 'device*':ab,ti OR 'framework*':ab,ti | 7,669,699 |
| #48 | 'social support':ab,ti | 65,811 |
| #49 | 'social media'/exp | 52,616 |
| #50 | 'social media':ab,ti OR 'facebook':ab,ti | 42,764 |
| #51 | 'social network'/exp | 26,424 |
| #52 | 'social network*':ab,ti OR 'network*, social':ab,ti | 29,210 |
| #53 | #30 OR #31 OR #32 OR #33 OR #34 OR #35 OR #36 OR #37 OR #38 OR #39 OR #40 OR #41 OR #42 OR #43 OR #44 OR #45 OR #46 OR #47 OR #48 OR #49 OR #50 OR #51 OR #52 | 8,256,938 |
| #54 | #29 AND #53 | 140,574 |
| #55 | #28 OR #54 | 141,997 |
| #56 | 'randomized controlled trial'/exp | 807,518 |
| #57 | 'controlled clinical trial'/exp | 988,189 |
| #58 | 'randomi?ed':ab | 1,092,377 |
| #59 | 'placebo':ab | 361,993 |
| #60 | 'clinical trial (topic)'/exp | 453,774 |
| #61 | 'randomly':ab | 564,916 |
| #62 | 'trial':ti | 419,324 |
| #63 | #56 OR #57 OR #58 OR #59 OR #60 OR #61 OR #62 | 2,383,122 |
| #64 | 'animal'/exp | 33,268,625 |
| #65 | 'human'/exp | 27,189,619 |
| #66 | #64 NOT #65 | 6,079,006 |
| #67 | #63 NOT #66 | 2,187,224 |
| #68 | 'exercise'/exp | 456,761 |
| #69 | 'exercise*':ab,ti OR 'active living':ab,ti OR 'active transport':ab,ti OR 'activities, physical':ab,ti OR 'activity, physical':ab,ti OR 'physical activities':ab,ti OR 'physical activity':ab,ti | 657,777 |
| #70 | 'exercise therapy':ab,ti | 6,495 |

Table S1. Continued.

| Search terms | | Results |
|---|---|---|
| #71 | 'fitness'/exp | 45,535 |
| #72 | 'fitness':ab,ti OR 'physical fitness':ab,ti | 112,833 |
| #73 | 'sedentary lifestyle'/exp | 21,427 |
| #74 | 'sedentary behavi*r?':ab,ti OR 'sedent*':ab,ti OR 'sitting time':ab,ti OR 'inactive':ab,ti OR 'inactivity':ab,ti OR 'walk*':ab,ti OR 'sedentary lifestyle':ab,ti | 411,806 |
| #75 | #68 OR #69 OR #70 OR #71 OR #72 OR #73 OR #74 | 1,185,665 |
| #76 | #24 AND #55 AND #67 AND #75 | 271 |
| #77 | #76 AND (2010:py OR 2011:py OR 2012:py OR 2013:py OR 2014:py OR 2015:py OR 2016:py OR 2017:py OR 2018:py OR 2019:py OR 2020:py OR 2021:py OR 2022:py OR 2023:py OR 2024:py) AND ([adult]/lim OR [aged]/lim OR [very elderly]/lim) AND 'article'/it | 84 |
| *Scopus* | | |
| #1 | TITLE-ABS-KEY ( "cardiovascular disease?" OR CVD OR "cardiocerebrovascular disease?" OR "cardiovascular event?" OR "heart disease?" OR stroke OR "myocardial infarction" OR MI OR "heart infarction" OR "myocardial ischemia" OR "myocardial ischaemia" OR "ischaemic heart disease?" OR "ischemic heart disease?" OR IHD OR "heart failure" OR "cardiac failure" OR "coronary artery disease?" OR CAD OR "coronary heart disease?" OR CHD OR "cerebral arterial disease?" OR "cerebral artery disease?" OR "cerebrovascular disorder?" OR "cerebrovascular disease?" OR "cerebral arterial disease?" OR "cerebral artery disease" OR CBVD OR "cerebral infarction" OR "cerebrovascular accident?" OR "cerebrovascular attack?" OR CVA OR "peripheral vascular disease?" OR "peripheral arterial disease?" OR "peripheral artery disease" OR PVD OR PAD ) | 2,020,450 |
| #2 | TITLE-ABS-KEY ( gamifi* OR exergam* OR "exer-gam*" ) | 19,321 |
| #3 | TITLE-ABS-KEY ( game? OR multiplayer OR player? OR playing OR play ) | 3,027,329 |
| #4 | TITLE-ABS-KEY ( telemedicine OR telehealth OR "e-health" OR "m-health" OR ehealth OR mhealth OR "mobile health" OR "digital health" OR application? OR app? OR online OR mobile OR internet OR "web based" OR web-based OR "phone?, smart" OR "smart phone?" OR "smartphone?" OR "cell phone?" OR cellphone? OR "mobile phone?" OR iphone OR android OR "iOS" OR "wearable electronic device?" OR "digital technolog*" OR website? OR digital* OR system* OR electronic* OR technolog* OR device? OR framework* OR "facebook" OR "social media" OR "social network" OR "network?, social" OR "social support" OR "support, social" OR "social networking" ) | 31,499,475 |
| #5 | #3 AND #4 | 1,261,049 |
| #6 | #2 OR #5 | 1,274,246 |
| #7 | TITLE-ABS-KEY ( "randomized controlled trial" OR "randomised controlled trial" ) | 1,013,876 |
| #8 | TITLE-ABS-KEY ( "controlled clinical trial" ) | 460,141 |
| #9 | ABS ( randomized OR randomised ) | 933,960 |
| #10 | ABS ( placebo ) | 274,103 |
| #11 | TITLE-ABS-KEY ( "clinical trials as topic" ) | 64,757 |
| #12 | ABS ( randomly ) | 707,546 |
| #13 | TITLE ( trial ) | 467,079 |
| #14 | #7 OR #8 OR #9 OR #10 OR #11 OR #12 OR #13 | 2,312,782 |
| #15 | TITLE-ABS-KEY ( animals OR animal ) | 8,359,934 |
| #16 | TITLE-ABS-KEY ( humans OR human ) | 26,684,655 |
| #17 | #15 AND NOT #16 | 5,456,511 |
| #18 | #14 AND NOT #17 | 2,144,727 |
| #19 | TITLE-ABS-KEY ( exercise* OR "active living" OR "active transport" OR "activit*, physical" OR "physical activit*" OR "exercise therapy" OR "physical fitness" OR fitness OR "sedentary behavior*" OR "sedentary behaviour*" OR sedent* OR "sitting time" OR inactive OR inactivity OR walk* ) | 1,729,652 |
| #20 | #1 AND #6 AND #18 AND #19 | 379 |
| #21 | #20 AND ( LIMIT-TO ( DOCTYPE , "ar" ) ) AND ( LIMIT-TO ( LANGUAGE , "English" ) ) AND PUBYEAR > 2009 AND PUBYEAR < 2025 | 210 |
| *Cochrane Library* | | |
| #1 | MeSH descriptor: [Cardiovascular Diseases] explode all trees | 154511 |
| #2 | MeSH descriptor: [Heart Diseases] explode all trees | 73755 |
| #3 | MeSH descriptor: [Stroke] explode all trees | 17122 |
| #4 | MeSH descriptor: [Myocardial Infarction] explode all trees | 15580 |
| #5 | MeSH descriptor: [Myocardial Ischemia] explode all trees | 38574 |
| #6 | MeSH descriptor: [Heart Failure] explode all trees | 14344 |
| #7 | MeSH descriptor: [Coronary Artery Disease] explode all trees | 9277 |
| #8 | MeSH descriptor: [Coronary Disease] explode all trees | 18903 |

Table S1. Continued.

| Search terms | | Results |
|---|---|---|
| #9 | MeSH descriptor: [Cerebrovascular Disorders] explode all trees | 24775 |
| #10 | MeSH descriptor: [Cerebral Arterial Diseases] explode all trees | 327 |
| #11 | MeSH descriptor: [Cerebral Infarction] explode all trees | 1637 |
| #12 | MeSH descriptor: [Peripheral Vascular Diseases] explode all trees | 5096 |
| #13 | MeSH descriptor: [Peripheral Arterial Disease] explode all trees | 2655 |
| #14 | ("cardiovascular disease":ti,ab,kw OR "cardiovascular diseases":ti,ab,kw OR "CVD":ti,ab,kw OR "cardiocerebrovascular disease":ti,ab,kw OR "cardiocerebrovascular diseases":ti,ab,kw OR "cardiovascular event":ti,ab,kw OR "cardiovascular events":ti,ab,kw OR "heart disease":ti,ab,kw OR "heart diseases":ti,ab,kw OR "stroke":ti,ab,kw OR "myocardial infarction":ti,ab,kw OR "MI":ti,ab,kw OR "heart infarction":ti,ab,kw OR "myocardial ischaemia":ti,ab,kw OR "myocardial ischemia":ti,ab,kw OR "ischaemic heart disease":ti,ab,kw OR "ischaemic heart diseases":ti,ab,kw OR "ischemic heart disease":ti,ab,kw OR "ischemic heart diseases":ti,ab,kw OR "IHD":ti,ab,kw OR "heart failure":ti,ab,kw OR "cardiac failure":ti,ab,kw OR "coronary artery disease":ti,ab,kw OR "coronary artery diseases":ti,ab,kw OR "CAD":ti,ab,kw OR "coronary heart disease":ti,ab,kw OR "coronary heart diseases" OR "coronary disease":ti,ab,kw OR "coronary diseases":ti,ab,kw OR "CHD":ti,ab,kw OR "cerebral arterial disease":ti,ab,kw OR "cerebral arterial diseases":ti,ab,kw OR "cerebral artery disease":ti,ab,kw OR "cerebral artery diseases":ti,ab,kw OR "cerebrovascular disorder":ti,ab,kw OR "cerebrovascular disorders":ti,ab,kw OR "CBVD":ti,ab,kw OR "cerebrovascular accident":ti,ab,kw OR "cerebrovascular accidents":ti,ab,kw OR "cerebrovascular disease":ti,ab,kw OR "cerebrovascular diseases":ti,ab,kw OR "cerebral infarction":ti,ab,kw OR "CVA":ti,ab,kw OR "peripheral vascular disease":ti,ab,kw OR "peripheral vascular diseases":ti,ab,kw OR "peripheral arterial disease":ti,ab,kw OR "peripheral arterial diseases":ti,ab,kw OR "peripheral artery disease":ti,ab,kw OR "peripheral artery diseases":ti,ab,kw OR "PVD":ti,ab,kw OR "PAD":ti,ab,kw) | 210010 |
| #15 | {OR #1-#14} | 273726 |
| #16 | MeSH descriptor: [Gamification] explode all trees | 26 |
| #17 | MeSH descriptor: [Exergaming] explode all trees | 71 |
| #18 | (gamification:ti,ab,kw OR exergaming:ti,ab,kw OR “exer-gaming”:ti,ab,kw) | 830 |
| #19 | {OR #16-#18} | 830 |
| #20 | (game:ti,ab,kw OR games:ti,ab,kw OR gamified:ti,ab,kw OR gaming:ti,ab,kw OR gameful*:ti,ab,kw OR multiplayer:ti,ab,kw OR player:ti,ab,kw OR players:ti,ab,kw OR playing:ti,ab,kw) | 15381 |
| #21 | MeSH descriptor: [Telemedicine] explode all trees | 4744 |
| #22 | MeSH descriptor: [Digital Health] explode all trees | 2 |
| #23 | MeSH descriptor: [Mobile Applications] explode all trees | 1898 |
| #24 | MeSH descriptor: [Internet] explode all trees | 6471 |
| #25 | MeSH descriptor: [Smartphone] explode all trees | 1128 |
| #26 | MeSH descriptor: [Cell Phone] explode all trees | 3421 |
| #27 | MeSH descriptor: [Wearable Electronic Devices] explode all trees | 979 |
| #28 | MeSH descriptor: [Digital Technology] explode all trees | 19 |
| #29 | MeSH descriptor: [Social Support] this term only | 4218 |
| #30 | MeSH descriptor: [Social Media] explode all trees | 562 |
| #31 | MeSH descriptor: [Social Networking] this term only | 213 |
| #32 | ("telemedicine":ti,ab,kw OR "telehealth":ti,ab,kw OR "e-Health":ti,ab,kw OR "m-Health":ti,ab,kw OR "eHealth":ti,ab,kw OR "mHealth":ti,ab,kw OR "mobile health":ti,ab,kw OR "digital health":ti,ab,kw OR "mobile application":ti,ab,kw OR "mobile applications":ti,ab,kw OR "applications":ti,ab,kw OR "application":ti,ab,kw OR app:ti,ab,kw OR apps:ti,ab,kw OR "online":ti,ab,kw OR "mobile":ti,ab,kw OR "internet":ti,ab,kw OR “web based”:ti,ab,kw OR "web-based":ti,ab,kw OR "phone, smart":ti,ab,kw OR "smart phones":ti,ab,kw OR "smartphones":ti,ab,kw OR "smart phone":ti,ab,kw OR "phones, smart":ti,ab,kw OR "smartphone":ti,ab,kw OR “cell phone”:ti,ab,kw OR "cell phones":ti,ab,kw OR "cellphone":ti,ab,kw OR "cellphones":ti,ab,kw OR "mobile phone":ti,ab,kw OR "mobile phones":ti,ab,kw OR Iphone:ti,ab,kw OR android:ti,ab,kw OR iOS:ti,ab,kw OR "wearable electronic device":ti,ab,kw OR "wearable electronic devices":ti,ab,kw OR "digital technology":ti,ab,kw OR "digital technologies":ti,ab,kw OR website?:ti,ab,kw OR digital*:ti,ab,kw OR system*:ti,ab,kw OR electronic*:ti,ab,kw OR technolog*:ti,ab,kw OR device?:ti,ab,kw OR framework*:ti,ab,kw OR "facebook":ti,ab,kw OR "networks, social":ti,ab,kw OR "social network":ti,ab,kw OR "network, social":ti,ab,kw OR "social networks":ti,ab,kw OR "social support":ti,ab,kw OR "support, social":ti,ab,kw OR "social networking":ti,ab,kw OR "social media":ti,ab,kw) | 500897 |
| #33 | {OR #21-#32} | 501710 |
| #34 | #20 AND #33 | 6311 |
| #35 | #19 OR #34 | 6840 |

Table S1. Continued.

| Search terms | | Results |
|---|---|---|
| #36 | MeSH descriptor: [Randomized Controlled Trial] explode all trees | 37 |
| #37 | MeSH descriptor: [Controlled Clinical Trial] explode all trees | 40 |
| #38 | (randomized:ab OR randomised:ab) | 844682 |
| #39 | placebo:ab | 348434 |
| #40 | MeSH descriptor: [Clinical Trials as Topic] explode all trees | 95883 |
| #41 | randomly:ab | 322266 |
| #42 | trial:ti | 432950 |
| #43 | {OR #36-#42} | 1329881 |
| #44 | (animal:ti,ab,kw OR animals:ti,ab,kw) | 40203 |
| #45 | (human:ti,ab,kw OR humans:ti,ab,kw) | 1297506 |
| #46 | #44 NOT #45 | 6000 |
| #47 | #43 NOT #46 | 1325639 |
| #48 | MeSH descriptor: [Exercise] explode all trees | 38282 |
| #49 | MeSH descriptor: [Exercise Therapy] explode all trees | 21433 |
| #50 | MeSH descriptor: [Physical Fitness] explode all trees | 4891 |
| #51 | (exercise:ti,ab,kw OR exercises:ti,ab,kw OR "active living":ti,ab,kw OR "active transport":ti,ab,kw OR "physical activity":ti,ab,kw OR "physical activities":ti,ab,kw OR "activity, physical":ti,ab,kw OR "activities, physical":ti,ab,kw) | 162352 |
| #52 | MeSH descriptor: [Sedentary Behavior] explode all trees | 1828 |
| #53 | ("sedentary behavior":ti,ab,kw OR "sedentary behaviors":ti,ab,kw OR "sedentary behaviour":ti,ab,kw OR "sedentary behaviours":ti,ab,kw OR sedent*:ti,ab,kw OR "sitting time":ti,ab,kw OR inactive:ti,ab,kw OR inactivity:ti,ab,kw OR walk*:ti,ab,kw OR "sedentary lifestyle":ti,ab,kw) | 58295 |
| #54 | {OR #48-#53} | 189508 |
| #55 | #15 AND #35 AND #47 AND #54 | 301 |
| #56 | with Publication Year from 2010 to 2024, with Cochrane Library publication date from Jan 2010 to Feb 2024, in Trials, Language: English | 290 |
| *CINAHL Plus with Full Text (EBSCOhost)* | | |
| S1 | (MH "Cardiovascular Diseases+") OR (MH "Heart Diseases+") OR (MH "Stroke+") OR (MH "Myocardial Infarction+") OR (MH "Myocardial Ischemia+") OR (MH "Heart Failure+") OR (MH "Coronary Arteriosclerosis") OR (MH "Coronary Disease+") OR (MH "Cerebrovascular Disorders+") OR (MH "Cerebral Arterial Diseases+") OR (MH "Cerebral Infarction") OR (MH "Peripheral Vascular Diseases+") | 684,649 |
| S2 | TI ( "cardiovascular disease*" OR CVD OR "cardiocerebrovascular disease*" OR "cardiovascular event*" OR "heart disease*" OR stroke OR "myocardial infarction" OR MI OR "heart infarction" OR "myocardial isch#mia" OR "isch#mic heart disease*" OR IHD OR "heart failure" OR "cardiac failure" OR "coronary artery disease*" OR CAD OR "coronary heart disease*" OR CHD OR "coronary arteriosclerosis" OR "coronary disease*" OR "cerebrovascular disorder*" OR "cerebrovascular disease*" OR CBVD OR "cerebral arterial disease*" OR "cerebral artery disease*" OR "cerebrovascular accident*" OR "cerebrovascular attack*" OR CVA OR "cerebral infarction" OR "peripheral vascular disease*" OR "peripheral arterial disease*" OR "peripheral artery disease*" OR PVD OR PAD ) OR AB ( "cardiovascular disease*" OR CVD OR "cardiocerebrovascular disease*" OR "cardiovascular event*" OR "heart disease*" OR stroke OR "myocardial infarction" OR MI OR "heart infarction" OR "myocardial isch#mia" OR "isch#mic heart disease*" OR IHD OR "heart failure" OR "cardiac failure" OR "coronary artery disease*" OR CAD OR "coronary heart disease*" OR CHD OR "coronary arteriosclerosis" OR "coronary disease*" OR "cerebrovascular disorder*" OR "cerebrovascular disease*" OR CBVD OR "cerebral arterial disease*" OR "cerebral artery disease*" OR "cerebrovascular accident*" OR "cerebrovascular attack*" OR CVA OR "cerebral infarction" OR "peripheral vascular disease*" OR "peripheral arterial disease*" OR "peripheral artery disease*" OR PVD OR PAD ) | 362,926 |
| S3 | S1 OR S2 | 808,607 |
| S4 | (MH "Gamification") OR (MH "Exergames") | 700 |
| S5 | TI ( gamification OR exergam* ) OR AB ( gamification OR exergam* ) | 1,167 |
| S6 | S4 OR S5 | 1,438 |
| S7 | TI ( game* OR gami* OR multiplayer OR player* OR playing ) OR AB ( game* OR gami* OR multiplayer OR player* OR playing ) | 54,544 |
| S8 | (MH "Telemedicine+") OR (MH "Telehealth+") OR (MH "Digital Health+") OR (MH "Mobile Applications") OR (MH "Internet+") OR (MH "Internet-Based Intervention") OR (MH "Smartphone") OR (MH "Cellular Phone+") OR (MH "Exercise Equipment and Supplies+") OR (MH "Wearable Sensors+") OR (MH "Digital Technology+") OR (MH "Support, Social") OR (MH "Social Media+") OR (MH "Social Networking") | 241,076 |

Table S1. Continued.

| Search terms | | Results |
|---|---|---|
| S9 | TI ( telemedicine OR telehealth OR e-Health OR m-Health OR eHealth OR mHealth OR "mobile health" OR "digital health" OR "mobile application*" OR application* OR app OR apps OR internet OR online OR mobile OR "web based" OR web-based OR "internet-based intervention" OR smartphone* OR "smart phone*" OR "phone*, smart" OR "cell phone*" OR "cellphone*" OR "mobile phone*" OR iPhone OR android OR iOS OR "wearable electronic device*" OR accelerometer* OR "fitness tracker*" OR pedometer* OR "wearable sensor*" OR "digital technolog*" OR website* OR digital* OR system* OR electronic* OR technolog* OR device* OR framework* OR "social support" OR "social media" OR "social network*" OR "network*, social" OR facebook OR twitter ) OR AB ( telemedicine OR telehealth OR e-Health OR m-Health OR eHealth OR mHealth OR "mobile health" OR "digital health" OR "mobile application*" OR application* OR app OR apps OR internet OR online OR mobile OR "web based" OR web-based OR "internet-based intervention" OR smartphone* OR "smart phone*" OR "phone*, smart" OR "cell phone*" OR "cellphone*" OR "mobile phone*" OR iPhone OR android OR iOS OR "wearable electronic device*" OR accelerometer* OR "fitness tracker*" OR pedometer* OR "wearable sensor*" OR "digital technolog*" OR website* OR digital* OR system* OR electronic* OR technolog* OR device* OR framework* OR "social support" OR "social media" OR "social network*" OR "network*, social" OR facebook OR twitter ) | 1,478,875 |
| S10 | S8 OR S9 | 1,591,013 |
| S11 | S7 AND S10 | 17,024 |
| S12 | S6 OR S11 | 17,758 |
| S13 | PT randomized controlled trial OR PT controlled clinical trial | 155,925 |
| S14 | AB randomi?ed OR placebo | 293,611 |
| S15 | (MH "Clinical Trials+") | 356,085 |
| S16 | AB randomly OR TI trial | 273,680 |
| S17 | S13 OR S14 OR S15 OR S16 | 599,115 |
| S18 | (MH "Animals+") | 104,478 |
| S19 | (MH "Human") | 2,776,302 |
| S20 | S18 NOT S19 | 94,704 |
| S21 | S17 NOT S20 | 594,253 |
| S22 | (MH "Exercise+") OR (MH "Physical Activity") OR (MH "Therapeutic Exercise+") OR (MH "Physical Fitness+") OR (MH "Life Style, Sedentary+") | 214,751 |
| S23 | TI ( exercise* OR "active living" OR "active transport" OR "physical activit*" OR "activit*, physical" OR "exercise therap*" OR "physical fitness" OR fitness OR "sedentary behavi#r*" OR sedent* OR "sitting time" OR inactive OR inactivity OR walk* ) OR AB ( exercise* OR "active living" OR "active transport" OR "physical activit*" OR "activit*, physical" OR "exercise therap*" OR "physical fitness" OR fitness OR "sedentary behavi#r*" OR sedent* OR "sitting time" OR inactive OR inactivity OR walk* ) | 277,742 |
| S24 | S22 OR S23 | 355,260 |
| S25 | S3 AND S12 AND S21 AND S24 | 69 |
| S26 | Limiters - Publication Date: 20100101-20240231; English Language; Human; Age Groups: All Adult | 30 |

Table S2. Characteristics of the included studies.

| Author, Year Country | Diagnosis | Model of delivery | T1 | T2 | Group | Sample | Age: mean (SD) | Male: n (%) | Content description of IG or CG | Outcomes |
|---|---|---|---|---|---|---|---|---|---|---|
| *Included in meta-analyses* | | | | | | | | | | |
| Xu et al., 2023 China | CHD | (a) WeChat applet "TahneeWeh" (b) Smartphone accelerometers | 3 months | 6 months | IG1 | 36 | 52.7 (10.8) | 28 (77.8) | Individual group: Participants received the gamification intervention based on BEP via the WeChat applet. The intervention embedded six BEP, which applied to four game elements: feedback, points, levels, and rewards. | Steps |
| | | | | | IG2 | 36 | 52.6 (10.8) | 30 (83.3) | Team group: In the team group, collaboration was added besides the four-game elements mentioned above. | |
| | | | | | CG | 36 | 53.7 (10.2) | 30 (83.3) | Participants received step goal setting and could see their progress on the WeChat applet. Participants received no other interventions. | |
| Gallagher et al., 2022 Australia | CHD | (a) The gamified mobile application "MyHeartMate" (b) Emails | 6 months | NA | IG | 194 | 60.9 (11.9) | 158 (81.4) | Participants received standard care, plus access to the MyHeartMate application, a brief (5 min) in-person information provision for uploading and logging in to the application for the first time, and the location of the freely available brief instructional video on the application goals and optimal use of the application functions. The application focused on promoting regular PA. | Total PA (MET-min/week) |
| | | | | | CG | 196 | 61.5 (11.2) | 162 (82.7) | Participants received standard care for CHD, which included medication prescriptions and risk factor reduction advice according to their medical provider's determination and the Heart Foundation's Managing My Heart Health information booklet. | |
| Patel et al., 2021 USA | ASCVD or a 10-year ASCVD risk score ≥ 7.5% | (a) Research technology platform "Way to Health" (b) Text messages (c) Wrist-worn wearable device | 4 months (a) 8-week introductory (b) 8-week maintenance | 6 months | IG1 | 99 | 57.4 (10.6) | 35 (35.4) | Participants in the gamification arms were randomly assigned to have step goals with assigned (2000-step increase from baseline) and gradual (step targets increase by 12.5% each week for 8 weeks to full goal by week 9) goals. Participants in the gamification arms received the same intervention implemented as follows. | (a) Steps (b) Minutes of MVPA |
| | | | | | IG2 | 99 | 57.5 (10.8) | 21 (21.2) | Gamification with assigned and immediate (strive for the goal beginning on day 1) goals. | |
| | | | | | IG3 | 106 | 58.7 (11.8) | 33 (31.1) | Gamification with choice (select a goal between 1000 and 3000 steps above baseline) and gradual goals. Participants in the choice arms could change their goal at any time during the study as long as it was within the provided range. | |
| | | | | | IG4 | 100 | 58 (10.9) | 39 (39.0) | Gamification with choice and immediate goals | |
| | | | | | CG | 96 | 61.1 (9.3) | 24 (25.0) | Participants received wearable devices but without establishing daily step goals, received daily feedback via text message on the previous day's step count each day, and received no other interventions. | |
| Radhakrishnan et al., 2021 USA | HF | (a) SCDG intervention application called "Heart Health Mountain" (b) Withings Go activity tracker | 3 months | NA | IG | 19 | 55-64: 20 (53%) 65-74: 12 (32%) | 10 (52.6) | The SCDG intervention group received sensors tracking weight monitoring (Withings Body smart weighing scale) and PA (Withings Go activity tracker) and played the SCDG application on a smartphone. The participants in the IG received standardized HF education embedded within the SCDG. | Steps |
| | | | | | CG | 19 | ⩾75: 6 (15%) | 10 (52.6) | The sensors-only control group received sensors tracking weight monitoring and PA only. The participants in the CG received the same standardized HF education in written format. | |
| Paldán et al., 2021 Germany | PAD | "TrackPAD" Application | 3 months | NA | IG | 19 | 64.6 (9.8) | 12 (63.2) | The intervention group included participants receiving standard care and additional mHealth-based self-tracking of their PA using TrackPAD. | Changes in 6MWD |
| | | | | | CG | 20 | 65.6 (7.7) | 9 (45) | The control group included participants with standard care and no further mobile intervention. | |

Table S2. Continued.

| Author, Year Country | Diagnosis | Model of delivery | T1 | T2 | Group | Sample | Age: mean (SD) | Male: n (%) | Content description of IG or CG | Outcomes |
|---|---|---|---|---|---|---|---|---|---|---|
| *Not included in meta-analyses* | | | | | | | | | | |
| Waddell et al., 2022 USA | Stroke | (a) Research technology platform “Way to Health”<br>(b) Emails<br>(c) Text messages<br>(d) Wearable device | 2 months | NA | IG | 17 | 57 (13.8) | 6 (35.3) | Participants were randomized to a gamification with social incentives arm received a wearable device worn on the unaffected wrist. They selected a step goal that was a 33%, 40%, or 50% increase from their baseline. The gamification arm engaged in an 8-week game with loss-framed points and levels to help participants achieve their step goal. Participants received daily (text message) and weekly (email) feedback describing their progress. Participants selected a support partner who identified 3 goals and received weekly email updates on the participants’ progress. | Steps |
| | | | | | CG | 17 | 61 (16.9) | 6 (35.3) | Received device feedback and no further intervention. | |

Abbreviations: T1, timepoint at post-intervention; T2, timepoint at the end of the pre-defined follow-up; SD, standard deviations; IG, intervention group; CG, control group; CHD, coronary heart disease; BEP, behavioral economics principles; NA, not applicable; PA, physical activity; MET, metabolic equivalent of task; USA, the United States of America; ASCVD, atherosclerotic cardiovascular disease; MVPA, moderate-to-vigorous physical activity; HF, heart failure; SCDG, sensor-controlled digital game; PAD, peripheral artery disease; mHealth, mobile health; 6MWD, 6-minute walking distance.

Table S3. Intervention characteristics with TIDieR headings.

| | Author, Year | | Xu et al., 2023 | Gallagher et al., 2022 | Patel et al., 2021 | Radhakrishnan et al., 2021 | Paldán et al., 2021 | Waddell et al., 2022 | Total *N* = 6 (100%) |
|---|---|---|---|---|---|---|---|---|---|
| Why? | | Theory | SDT, BEP | SCT | GST, BEP | FBM | | BEP | 5 (83.3%) |
| What? | 1. Goal setting | 1. Goals | ✓ | | ✓ | ✓ | ✓ | ✓ | 5 (83.3%) |
| | 2. Capacity to overcome challenges | 2. Challenges | | ✓ | | ✓ | ✓ | | 3 (50%) |
| | | 3. Points | ✓ | | ✓ | | | ✓ | 3 (50%) |
| | | 4. Levels | ✓ | | ✓ | | | ✓ | 3 (50%) |
| | 3. Providing feedback on performance | 5. Feedback | ✓ | ✓ | ✓ | ✓ | | ✓ | 5 (83.3%) |
| | 4. Reinforcement | 6. Rewards | ✓ | ✓ | | ✓ | ✓ | | 4 (66.7%) |
| | 5. Compare progress | 7. Progress bars | ✓ | ✓ | | ✓ | ✓ | | 4 (66.7%) |
| | | 8. Leaderboards | | ✓ | | ✓ | ✓ | | 3 (50%) |
| | 6. Social connectivity | 9. Social support | | | | | | ✓ | 1 (16.7%) |
| | | 10. Collaboration | ✓ | | | | | | 1 (16.7%) |
| | 7. Fun and playfulness | 11. Avatars | | ✓ | | ✓ | | | 2 (33.3%) |
| How? | | Smartphone app | ✓ | ✓ | | ✓ | ✓ | | 4 (66.7%) |
| | | Website | | | ✓ | | | ✓ | 2 (33.3%) |
| | | Emails | | ✓ | | | | ✓ | 2 (33.3%) |
| | | Text messages | | | ✓ | | | ✓ | 2 (33.3%) |
| | | Wearable device | | | ✓ | ✓ | | ✓ | 4 (66.7%) |
| How long? | Intervention period | Short: < 3 months | | | | | | ✓ | 1 (16.7%) |
| | | Med: ≥ 3 months | ✓ | | ✓ | ✓ | ✓ | | 4 (66.7%) |
| | | Long: ≥ 6 months | | ✓ | | | | | 1 (12.5%) |
| Tailoring? | | Personalized goals | ✓ | | | ✓ | ✓ | ✓ | 4 (66.7%) |
| How well? | Attrition (post-intervention) | Low: 0-12.99% | ✓ | | ✓ | | | ✓ | 3 (50%) |
| | | Med: 13-26% | | | | ✓ | ✓ | | 2 (33.3%) |
| | | High: > 26% | | ✓ | | | | | 1 (16.7%) |

Abbreviations: SDT, self-determination theory; BEP, behavioral economics principles; SCT, social cognitive theory; GST, goal-setting theory; FBM, Fogg’s behavioral model.

Table S4. Details of the gamification intervention.

| Game design elements | Xu et al., 2023 | Gallagher et al., 2022 | Patel et al., 2021 | Radhakrishnan et al., 2021 | Paldán et al., 2021 | Waddell et al., 2022 |
|---|---|---|---|---|---|---|
| *Goal setting: Committing to achieve a goal* | | | | | | |
| Goals | Personalized daily step goals will be set based on patients' baseline daily step counts, and the goals will increase gradually from the baseline by 15% each week during the first 6 weeks and then remain fixed during the last 6 weeks. | NA | (a) Assigned goals: 2000-step increase from baseline<br>(b) Choice goals: select a goal between 1000 and 3000 steps above the baseline<br>(c) Immediate goals: strive for the goal beginning on day 1<br>(d) Gradual goals: step targets increase by 12.5% each week for 8 weeks to full goal by week 9 | The step goals for all participants were set based on their preferences as well as the nursing research assistant's assessment of their physical health status. The step goal in the SCDG ranged from 3000 to 15,000 steps. | The participants set their weekly goal of SET units at the beginning of each week. The app suggested a new weekly goal using an internal algorithm based on a user's SET unit's completion rate during the previous week. | Participants selected a step goal of a 33%, 40%, or 50% increase from their baseline. |
| *Capacity to overcome challenges: Growth, learning, and development* | | | | | | |
| Challenges | NA | Participants were provided with incremental challenges to promote sustained behaviour change. | NA | … problem-solving challenge (e.g., quizzes) … | Nest the challenge into a badge. For example, completing the training challenge for seven consecutive days can unlock the "You trained seven days in a row" badge. | NA |
| Points | The patients will receive 140 points every Monday (20 for each day). If the patients reach the target step count, no points will be deducted; if not, 20 points will be deducted. | NA | At the beginning of each week, the participant received 70 points. If the participant did not achieve their step goal on a given day, they lost 10 points from their balance. | NA | NA | Participants will receive 70 points in their virtual account at the start of each week. If they fail to meet their daily step goal, 10 points will be deducted from their account. |
| Levels | The patient is set to the middle of the 5 levels. If the patient's total score is less than 80 points in a week, the level will drop; otherwise, the level will rise. | NA | At the end of each week, participants moved up a level if they had at least 40 points. If not, participants dropped a level. All participants began at the middle of the 5 levels. | NA | NA | There are 5 levels, and all participants will start at the middle level. At the end of each week, participants who do not have 40 points will drop a level. |
| *Providing feedback on performance: Receiving constant feedback through the experience* | | | | | | |
| Feedback | Patients in the two intervention groups will receive weekly feedback on their progress. | …receiving congratulatory emails personalized through participant's name and specific achievement. | …provided daily feedback via text message on their progress. | e.g., Oh no, we didn't exercise. We can do better! | NA | Participants will receive a daily text message informing them if they met their step goal the previous day. |
| *Reinforcement: Gaining rewards, avoiding punishments* | | | | | | |
| Rewards | At the end of the intervention, if the patients' level is diamond, they will be rewarded with a small prize. | Earning coins for completing health-related challenges, monitoring health data, correctly answering quizzes and receiving congratulatory emails personalized by participant's name and specific achievement. | NA | Game rewards: Coins earned by solving in-game challenges help purchase healthy recipes or accessories for the game avatar. | Each user's personal progress was recorded to unlock achievement medals (e.g., a notable increase in PA, activity performed during public holidays, or successes like an increase in performed SET units per week). | NA |

Table S4. Continued.

| Game design elements | Xu et al., 2023 | Gallagher et al., 2022 | Patel et al., 2021 | Radhakrishnan et al., 2021 | Paldán et al., 2021 | Waddell et al., 2022 |
|---|---|---|---|---|---|---|
| *Compare progress: Monitoring progress with self and others* | | | | | | |
| Progress bars | Patients can see their daily progress toward their goals using a circular dial on the WeChat applet. | Monitor and track performance: Encourage awareness and goal attainment by regularly entering health data (exercise), which is then displayed on graphs. | NA | We selected the Withings Go tracker and used a clock schema, a familiar interface for most adults, to represent the user's daily PA. | Weekly progress: Summarize the progress of the current week.<br>The time bar shows the elapsed time of the current SET unit while training. | NA |
| Leaderboards | NA | Engagement and participation in health challenges through a community leader board (e.g., Users coin tally is ranked against the entire community and their friends). | NA | Figure 3 shows the leaderboard. | The leaderboard contained four different categories. The different leaderboards showed individual placements compared to other users using TrackPAD. | NA |
| *Social connectivity: Interacting with other people* | | | | | | |
| Collaboration | Points will not be deducted if both the patient and team member meet the goal. If the patient meets the goal but the team member does not, 10 points will be deducted. If the entire team does not meet the goal, 20 points will be deducted. | NA | NA | NA | NA | NA |
| Social support | NA | NA | NA | NA | NA | Participants were asked to designate a support partner who will receive weekly progress updates. Prior to the intervention, a three-way conference call was scheduled with the participant, support partner, and research team to explain the rules and develop 3 support strategies. |
| *Fun and playfulness: Paying out an alternative reality* | | | | | | |
| Avatars | NA | Gamification is centered on a cartoon heart avatar as a virtual representation of the user's health. The avatar personalizes the effects of health behaviour: a virtual heart that flourishes when provided healthy food, exercise, relaxation, and medications purchased through earned coins. | NA | The Heart Health Mountain SCDG presented a narrative in which the older adult player helps an avatar to climb a mountain in a forested area. As the avatar climbs the heart mountain …<br>… helped purchase healthy recipes or accessories for the game avatar. | NA | NA |

Abbreviations: NA, not applicable; SCDG, sensor-controlled digital game; SET, supervised exercise training; PA, physical activity; PAD, peripheral arterial disease.

Table S5. TIDieR reporting in each study.

| Author, Year | Xu et al., 2023 | Gallagher et al., 2022 | Patel et al., 2021 | Radhakrishnan et al., 2021 | Paldán et al., 2021 | Waddell et al., 2022 | Total *N* = 6 (100%) |
|---|---|---|---|---|---|---|---|
| 1. Brief name | Y | Y | Y | Y | Y | Y | 6 (100%) |
| 2. Why | Y | Y | Y | Y | Y | Y | 6 (100%) |
| 3. What (materials) | N | ? | N | Y | ? | N | 1 (16.7%) |
| 4. What (procedures) | Y | Y | Y | Y | Y | Y | 6 (100%) |
| 5. Who provided | ? | Y | ? | ? | ? | ? | 1 (16.7%) |
| 6. How | Y | Y | Y | Y | Y | Y | 6 (100%) |
| 7. Where | ? | ? | ? | Y | ? | ? | 1 (16.7%) |
| 8. When and how much | Y | Y | Y | Y | Y | Y | 6 (100%) |
| 9. Tailoring | Y | N | N | Y | Y | Y | 4 (66.7%) |
| 10. Modifications | N | Y | N | N | N | N | 1 (16.7%) |
| 11. How well (planned) | N | Y | N | Y | Y | N | 3 (50%) |
| 12. How well (actual) | N | Y | N | Y | Y | N | 3 (50%) |
| Total *N* = 12 (100%) | 6 (50%) | 9 (75%) | 5 (41.7%) | 10 (83.3%) | 8 (66.7%) | 6 (50%) | |

Abbreviations: Y, yes; N, no; ?, unclear.

Table S6. Outcomes data of the included studies.

| Author, Year | Measurement tool | Measurement method | Units of measurement | Group | T0 | | T1 | | | T2 | | |
|---|---|---|---|---|---|---|---|---|---|---|---|---|
| *Included in the meta-analysis, data presented as mean ± SD* | | | | | | | | | | | | |
| Xu et al., 2023 | Smartphone accelerometers | Objective | Steps/day | IG1 | 5796 | 2900 | 7159 | 3320 | *P* = .009 | 6390 | 2894 | *P* = .002 |
| | | | | IG2 | 6133 | 4200 | 6980 | 3571 | *P* = .12 | 6049 | 3319 | *P* = .78 |
| | | | | CG | 5866 | 2152 | 6241 | 1935 | | 5642 | 1631 | |
| Patel et al., 2021 | Wrist-worn wearable device (Fitbit Alta or Fitbit Inspire; Fitbit Inc) | Objective | Steps/day | IG1 | 5792 | 2352 | 6828 | 2804 | *P* = .04 | 6891 | 2850 | *P* = .01 |
| | | | | IG2 | 5639 | 2339 | 6610 | 2859 | *P* = .08 | 6463 | 2750 | *P* = .12 |
| | | | | IG3 | 5715 | 2160 | 6728 | 2697 | *P* = .05 | 6548 | 2630 | *P* = .06 |
| | | | | IG4 | 5895 | 2190 | 7681 | 3033 | *P* < .001 | 7578 | 3203 | *P* < .001 |
| | | | | CG | 5847 | 2330 | 6257 | 2512 | | 6193 | 2602 | |
| | Estimated by totaling the number of minutes per day that had a step cadence of 100 steps or more per minute. | Objective | Minutes/day (MVPA) | IG1 | 5.8 | 7.6 | 9.2 | 8.3 | *P* = .05 | 8.4 | 7.6 | *P* = .18 |
| | | | | IG2 | 5.8 | 6.9 | 9.9 | 9.6 | *P* = .008 | 9.6 | 9.4 | *P* = .02 |
| | | | | IG3 | 5.3 | 6.5 | 9.1 | 10.4 | *P* = .03 | 8.2 | 9.6 | *P* = .11 |
| | | | | IG4 | 7 | 7.5 | 11.9 | 12.9 | *P* < .001 | 11.0 | 13.0 | *P* = .004 |
| | | | | CG | 6.8 | 8.3 | 7.6 | 9.0 | | 7.5 | 9.5 | |
| Radhakrishnan et al., 2021 | Withings Go activity tracker | Objective | Steps/day | IG | NA | NA | 2887 | 2821 | NA | | | |
| | | | | CG | NA | NA | 2541 | 1604 | | | | |
| *Included in the meta-analysis, data presented as MD (SD of the Difference)* | | | | | | | | | | | | |
| Paldán et al., 2021 | 6MWT | Objective | Meters | IG | NA | NA | 83.0 | 72.2 | *P* = .01 | | | |
| | | | | CG | NA | NA | -38.8 | -22.0 | | | | |
| *Included in the meta-analysis, data presented as Median (IQR)* | | | | | | | | | | | | |
| Gallagher et al., 2022 | GPAQ | Self-reported | MET-min/week (Total PA) | IG | 1720 | 900, 3720 | 1860 | 840, 3600 | *P* = .06 | | | |
| | | | | CG | 1960 | 960, 4200 | 1570 | 800, 3000 | | | | |
| *Not included in meta-analysis, lack of post-intervention data, data presented as mean ± SD* | | | | | | | | | | | | |
| Waddell et al., 2022 | Wearable device (Fitbit) | Objective | Steps/day | IG | 4284 | 1316 | NA | NA | *P* = .01 | | | |
| | | | | CG | 4312 | 1515 | NA | NA | | | | |

Abbreviations: T0: timepoint at baseline; T1: timepoint at post-intervention; T2: timepoint at the end of the pre-defined follow-up; SD: standard deviations; IG: intervention group; CG: control group; MVPA: moderate-to-vigorous physical activity; MD: mean differences; 6MWT: 6-minute walking test; GPAQ: Global Physical Activity Questionnaire; MET: metabolic equivalent of task; PA: physical activity; IQR: interquartile range.

Table S7. Details of risk of bias assessment of included studies.

| Domains and Signaling questions | Response | | | | | |
|---|---|---|---|---|---|---|
| | Xu et al., 2023 | Gallagher et al., 2022 | Patel et al., 2021 | Radhakrishnan et al., 2021 | Paldán et al., 2021 | Waddell et al., 2022 |
| *Domain 1. Randomization process* | | | | | | |
| 1.1 Was the allocation sequence random? | Y | Y | Y | Y | Y | Y |
| 1.2 Was the allocation sequence concealed until participants were enrolled and assigned to interventions? | Y | Y | Y | Y | Y | Y |
| 1.3 Did baseline differences between intervention groups suggest a problem with the randomization process? | N | N | N | N | N | N |
| Risk of bias judgement | Low | Low | Low | Low | Low | Low |
| *Domain 2. Deviations from intended interventions* | | | | | | |
| 2.1 Were participants aware of their assigned intervention during the trial? | Y | Y | Y | Y | Y | Y |
| 2.2 Were carers and people delivering the interventions aware of participants' assigned intervention during the trial? | Y | N | Y | Y | Y | Y |
| 2.3 If Y/PY/NI to 2.1 or 2.2: Were there deviations from the intended intervention that arose because of the trial context? | N | N | N | N | N | N |
| 2.4 If Y/PY to 2.3: Were these deviations likely to have affected the outcome? | NA | NA | NA | NA | NA | NA |
| 2.5 If Y/PY/NI to 2.4: Were these deviations from intended intervention balanced between groups? | NA | NA | NA | NA | NA | NA |
| 2.6 Was an appropriate analysis used to estimate the effect of assignment to intervention? | Y | Y | Y | Y | N | Y |
| 2.7 If N/PN/NI to 2.6: Was there potential for a substantial impact (on the result) of the failure to analyse participants in the group to which they were randomized? | NA | NA | NA | NA | N | NA |
| Risk of bias judgement | Low | Low | Low | Low | Some concerns | Low |
| *Domain 3. Missing outcome data* | | | | | | |
| 3.1 Were data for this outcome available for all, or nearly all, participants randomized? | Y | Y | Y | Y | N | Y |
| 3.2 If N/PN/NI to 3.1: Is there evidence that the result was not biased by missing outcome data? | NA | NA | NA | NA | PN | NA |
| 3.3 If N/PN to 3.2: Could missingness in the outcome depend on its true value? | NA | NA | NA | NA | N | NA |
| 3.4 If Y/PY/NI to 3.3: Is it likely that missingness in the outcome depended on its true value? | NA | NA | NA | NA | NA | NA |
| Risk of bias judgement | Low | Low | Low | Low | Low | Low |
| *Domain 4. Measurement of the outcome* | | | | | | |
| 4.1 Was the method of measuring the outcome inappropriate? | N | N | N | N | N | N |
| 4.2 Could measurement or ascertainment of the outcome have differed between intervention groups? | N | N | N | N | N | N |
| 4.3 If N/PN/NI to 4.1 and 4.2: Were outcome assessors aware of the intervention received by study participants? | N | N | N | N | N | N |
| 4.4 If Y/PY/NI to 4.3: Could assessment of the outcome have been influenced by knowledge of intervention received? | NA | NA | NA | NA | NA | NA |
| 4.5 If Y/PY/NI to 4.4: Is it likely that assessment of the outcome was influenced by knowledge of intervention received? | NA | NA | NA | NA | NA | NA |
| Risk of bias judgement | Low | Low | Low | Low | Low | Low |
| *Domain 5. Selection of the reported result* | | | | | | |
| 5.1 Were the data that produced this result analysed in accordance with a prespecified analysis plan that was finalized before unblinded outcome data were available for analysis? | Y | Y | Y | Y | Y | Y |
| 5.2 …multiple eligible outcome measurements (e.g. scales, definitions, time points) within the outcome domain? | N | N | N | N | N | N |
| 5.3 …multiple eligible analyses of the data? | N | N | N | N | N | N |
| Risk of bias judgement | Low | Low | Low | Low | Low | Low |
| *Overall bias* | | | | | | |
| Risk of bias judgement | Low | Low | Low | Low | Some concerns | Low |

Abbreviations: Y: yes; PY: probably yes; PN: probably no; N: no; NI: no information; NA: not applicable.

Table S8. Best 5 models (model selection table).

| | (Intercept) | Avatars | Challenges | Collaboration | Feedback | Goals | Leaderboards | Levels | Points | Progress bars | Rewards | df | logLik | AICc | delta | weight |
|---|---|---|---|---|---|---|---|---|---|---|---|---|---|---|---|---|
| 25 | + | | | | + | + | | | | | | 4 | 4.498 | 4.0 | 0.00 | 0.062 |
| 9 | + | | | | + | | | | | | | 3 | 1.177 | 6.3 | 2.31 | 0.020 |
| 41 | + | | | | + | | + | | | | | 4 | 2.870 | 7.3 | 3.26 | 0.012 |
| 105 | + | | | | + | | + | + | | | | 4 | 2.870 | 7.3 | 3.26 | 0.012 |
| 169 | + | | | | + | | + | | + | | | 4 | 2.870 | 7.3 | 3.26 | 0.012 |

Table S9. Summary of the quality of the evidence for gamification versus control.

| Certainty assessment | | | | | | | № of patients | | Effect | Certainty |
|---|---|---|---|---|---|---|---|---|---|---|
| № of studies | Study design | Risk of bias | Inconsistency | Indirectness | Imprecision | Other considerations | gamification | control | Absolute (95% CI) | |
| Short-term PA (after sensitivity analysis) | | | | | | | | | | |
| 12 | randomized trials | not serious | not serious | not serious | not serious | none | 1023 | 366 | SMD 0.32 SD higher (0.19 higher to 0.45 higher) | ⊕⊕⊕⊕ High |
| Pre-defined follow-up PA | | | | | | | | | | |
| 10 | randomized trials | not serious | not serious | not serious | not serious | none | 880 | 228 | SMD 0.20 SD higher (0.12 higher to 0.29 higher) | ⊕⊕⊕⊕ High |
| Daily steps | | | | | | | | | | |
| 7 | randomized trials | not serious | not serious | not serious | not serious | none | 491 | 148 | MD 696.96 higher (327.80. higher to 1066.12 higher) | ⊕⊕⊕⊕ High |

Abbreviations: CI: confidence interval; PA: physical activity; SMD: standardized mean difference; SD: standard deviation.

Table S10. Summary of the application of the principles of behavioral economics.

| Principles | Pre-commitment | Fresh Start Effect | Prospect Theory/Loss Eversion | Goal Gradients | Social Norms |
|---|---|---|---|---|---|
| Xu et al., 2023 | ✓ | ✓ | ✓ | ✓ | ✓ |
| Patel et al., 2021 | | ✓ | ✓ | ✓ | |
| Waddell et al., 2022 | ✓ | ✓ | ✓ | ✓ | |
| Game design elements | Goals | Points | Points | Levels | Collaboration |
| Application | Participants were asked to sign an electronic commitment pledge, promising to strive to achieve their goals during the study period. | Participants would receive a new set of points at the beginning of each week. | Participants were informed their points would remain the same if they met the daily step goal; otherwise, they would be deducted. | At the start of the study, each participant began at the middle level. If the goal were achieved at least four days a week, the level would increase; otherwise, it would decrease. | The changes in points, level up and down, and reward or not depended on each participant within the team. |
| Implications | Requesting individuals to commit to desired behaviors or goals, especially publicly, is a method to bridge the intention-behavior gap, as humans strive for consistency within themselves. | People are more likely to achieve their goals immediately after prominent temporal landmarks (such as the start of a new week, month, year, or semester), which delineate the passage of time, categorizing past imperfections as belonging to the previous period, prompting individuals to view their lives from a broader perspective, thus motivating aspirational behavior. | Losses are believed to have a greater impact on individuals than gains. Framing incentives in terms of losses to encourage increased PA may be more pronounced, as people are more motivated to avoid the sorrow of loss rather than seek the pleasure of gain. | "Goal Gradients" refers to the tendency to increase effort as one approaches goal completion, i.e., to mobilize more resources to carry out instrumental behavior. The advancement in levels signifies progress toward the overall goal. As participants gradually elevate their levels to approach the overarching goal, their level of effort intensifies, giving them more motivation to achieve it. | "Social Norms" refers to the behavioral expectations or rules of a society or group that individuals within the group strive to adhere to. Participants' performance was closely linked to the team's status, and humans have an ingrained desire to maintain a positive social image. Therefore, participants would measure themselves against predetermined social norms, influencing their PA behavior. |

Abbreviations: PA: physical activity.

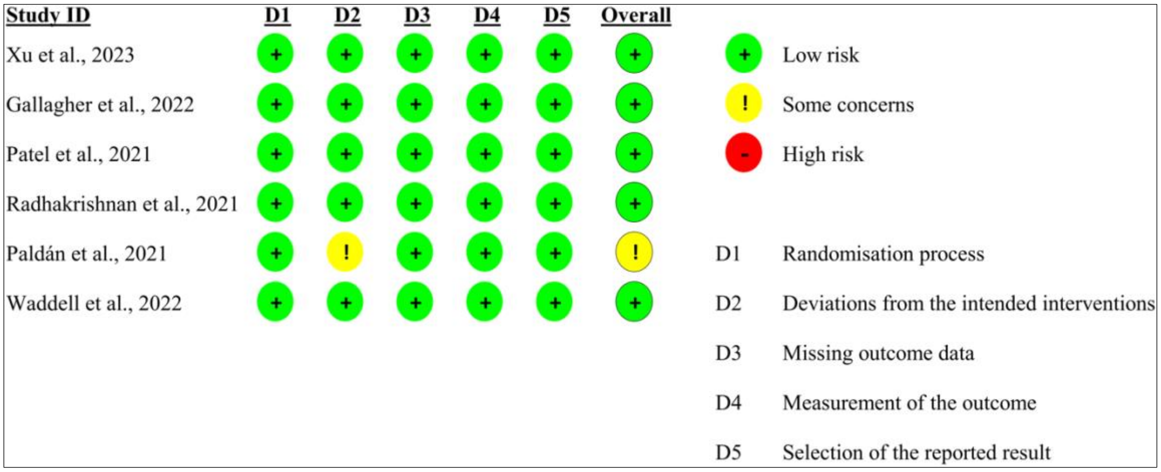


Figure S1. Risk of bias.

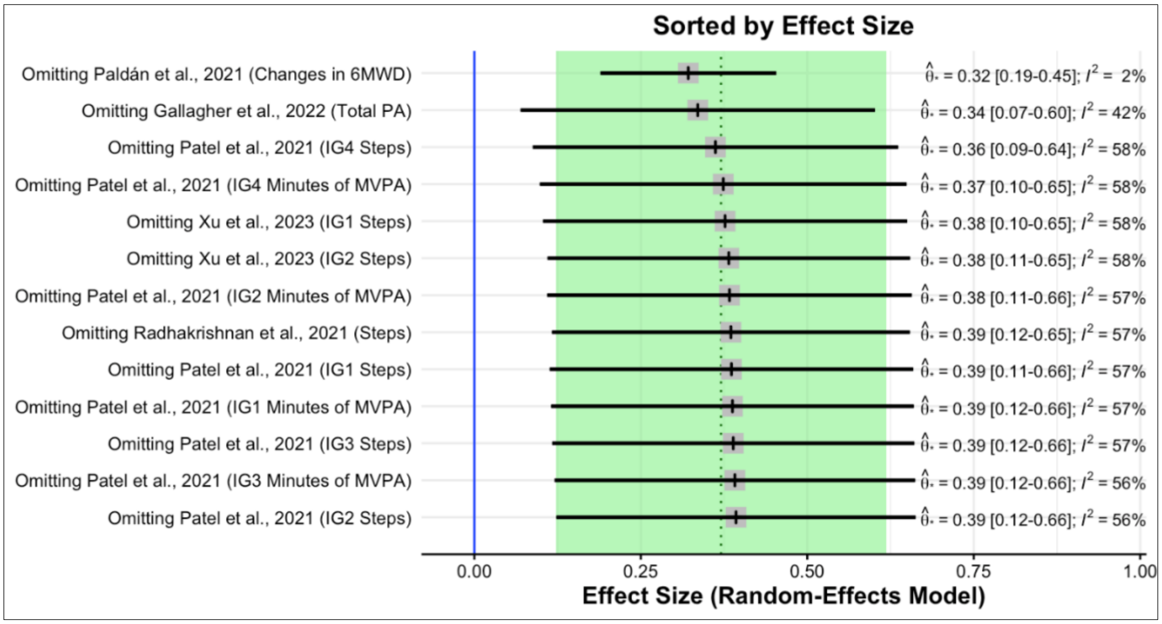


Figure S2. Forest plot representing Leave-one-out analyses ordered by effect size (Hedge’s g; low to high).

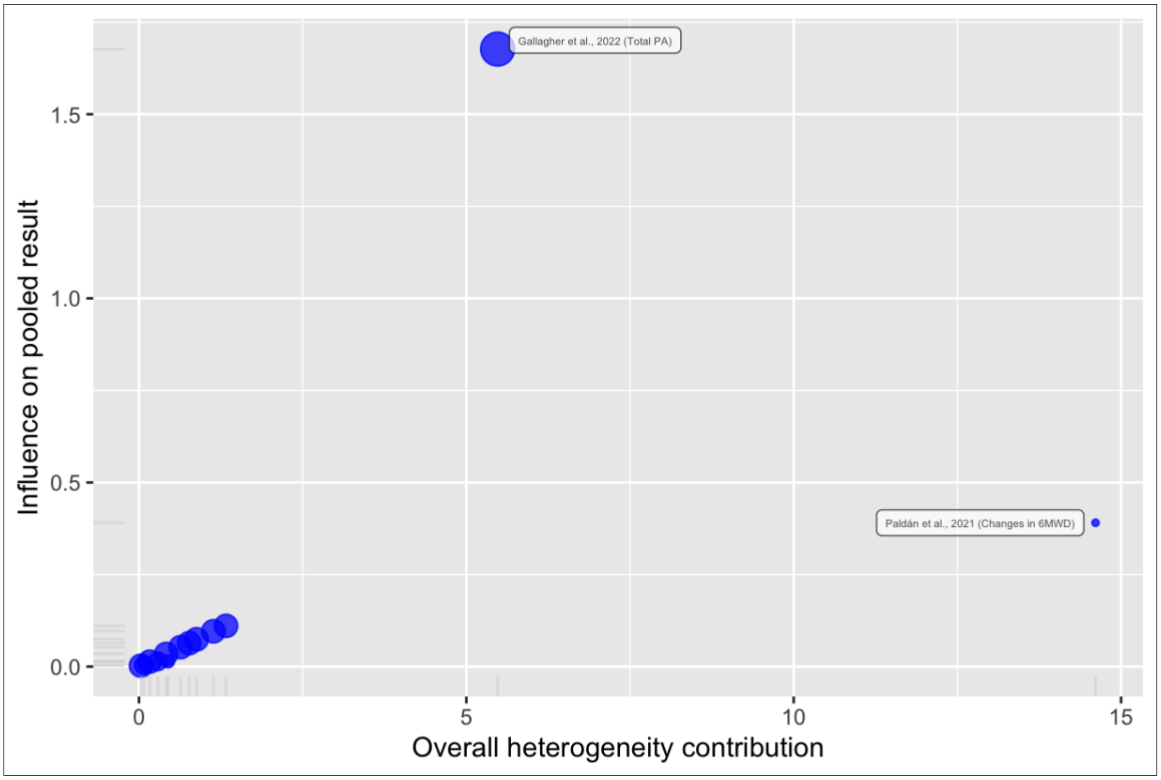


Figure S3. Baujat plot representing each study's contribution to the overall heterogeneity (measured by Cochran's Q) as a function of its influence on the pooled effect size.

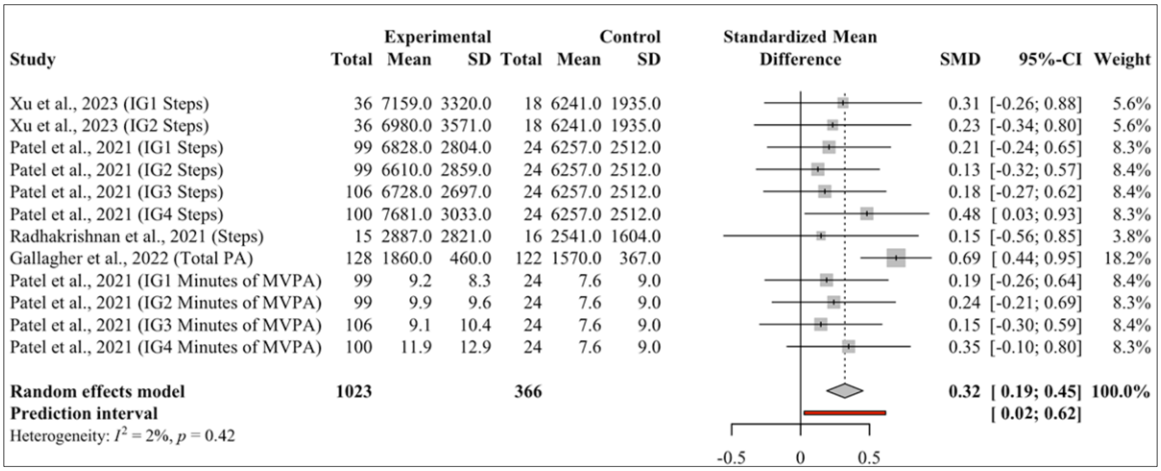

| Study | Experimental Total | Experimental Mean | Experimental SD | Control Total | Control Mean | Control SD | SMD | 95%-CI | Weight |
|---|---|---|---|---|---|---|---|---|---|
| Xu et al., 2023 (IG1 Steps) | 36 | 7159.0 | 3320.0 | 18 | 6241.0 | 1935.0 | 0.31 | [-0.26; 0.88] | 5.6% |
| Xu et al., 2023 (IG2 Steps) | 36 | 6980.0 | 3571.0 | 18 | 6241.0 | 1935.0 | 0.23 | [-0.34; 0.80] | 5.6% |
| Patel et al., 2021 (IG1 Steps) | 99 | 6828.0 | 2804.0 | 24 | 6257.0 | 2512.0 | 0.21 | [-0.24; 0.65] | 8.3% |
| Patel et al., 2021 (IG2 Steps) | 99 | 6610.0 | 2859.0 | 24 | 6257.0 | 2512.0 | 0.13 | [-0.32; 0.57] | 8.4% |
| Patel et al., 2021 (IG3 Steps) | 106 | 6728.0 | 2697.0 | 24 | 6257.0 | 2512.0 | 0.18 | [-0.27; 0.62] | 8.4% |
| Patel et al., 2021 (IG4 Steps) | 100 | 7681.0 | 3033.0 | 24 | 6257.0 | 2512.0 | 0.48 | [ 0.03; 0.93] | 8.3% |
| Radhakrishnan et al., 2021 (Steps) | 15 | 2887.0 | 2821.0 | 16 | 2541.0 | 1604.0 | 0.15 | [-0.56; 0.85] | 3.8% |
| Gallagher et al., 2022 (Total PA) | 128 | 1860.0 | 460.0 | 122 | 1570.0 | 367.0 | 0.69 | [ 0.44; 0.95] | 18.2% |
| Patel et al., 2021 (IG1 Minutes of MVPA) | 99 | 9.2 | 8.3 | 24 | 7.6 | 9.0 | 0.19 | [-0.26; 0.64] | 8.4% |
| Patel et al., 2021 (IG2 Minutes of MVPA) | 99 | 9.9 | 9.6 | 24 | 7.6 | 9.0 | 0.24 | [-0.21; 0.69] | 8.3% |
| Patel et al., 2021 (IG3 Minutes of MVPA) | 106 | 9.1 | 10.4 | 24 | 7.6 | 9.0 | 0.15 | [-0.30; 0.59] | 8.4% |
| Patel et al., 2021 (IG4 Minutes of MVPA) | 100 | 11.9 | 12.9 | 24 | 7.6 | 9.0 | 0.35 | [-0.10; 0.80] | 8.3% |
| **Random effects model** | **1023** | | | **366** | | | **0.32** | **[ 0.19; 0.45]** | **100.0%** |
| **Prediction interval** | | | | | | | | **[ 0.02; 0.62]** | |
| Heterogeneity: $I^2 = 2\%$, $p = 0.42$ | | | | | | | | | |



Figure S4. Forest plot for the effect of short-term physical activity after sensitivity analysis.

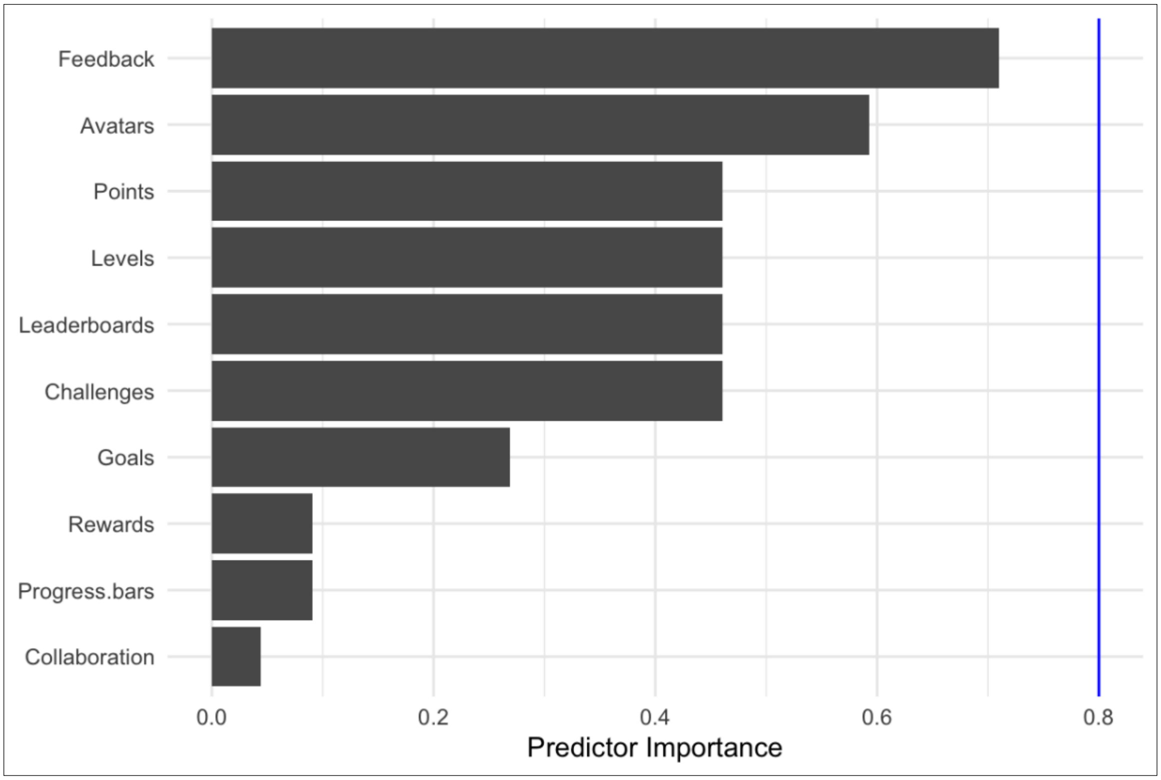

Figure S5. Predictor importance plot of game design elements.
